# One‐Step Drug Screening System Utilizing Electrophysiological Activity in Multiple Brain Organoids

**DOI:** 10.1002/advs.202504913

**Published:** 2025-09-12

**Authors:** Hyogeun Shin, Yeonjoo An, Ju‐Hyun Lee, Ji Hun Kim, Renuka Prasad, Keun‐Tae Kim, Hoon‐Chul Kang, Woong Sun, Seung‐Woo Cho, Il‐Joo Cho

**Affiliations:** ^1^ School of Electronic and Electrical Engineering College of IT Engineering Kyungpook National University Daegu 41566 Republic of Korea; ^2^ Department of Biotechnology Yonsei University Seoul 03722 Republic of Korea; ^3^ Cellartgen Seoul 03722 Republic of Korea; ^4^ Center for Brain Technology Brain Science Institute Korea Institute of Science and Technology (KIST) Seoul 02792 Republic of Korea; ^5^ Division of Pediatric Neurology Department of Pediatrics Severance Children's Hospital Yonsei University College of Medicine Seoul 03722 Republic of Korea; ^6^ Department of Convergence Medicine College of Medicine Korea University Seoul 02841 Republic of Korea; ^7^ Department of Anatomy Brain Korea 21 Plus Program for Biomedical Science Korea University Seoul 02841 Republic of Korea; ^8^ Center for Nanomedicine Institute for Basic Science (IBS) Seoul 03722 Republic of Korea

**Keywords:** 3D electrode array, brain organoid, drug screening, electrophysiological activity, microfluidic chip

## Abstract

Human‐induced pluripotent stem cell (iPSC)‐derived brain organoids have attracted significant attention as promising models for drug screening owing to their remarkable resemblance to the human brain. The advent of disease model organoids, derived from patient's cells, has further elevated expectations for drug screening and personalized medicine. Nevertheless, the absence of a comprehensive platform for administering drugs and assessing their efficacy based on functional changes in brain organoids has remained a challenge. In this study, we introduce a one‐step drug screening system designed designed to investigate functional changes induced by diverse drug doses in multiple brain organoids, utilizing electrophysiological signal measurements. Our system comprises a specialized culture chamber with a microfluidic chip capable of accommodating 10 organoids and delivering varying doses of two drugs to each organoid. Additionally, we integrate a three dimentional microelectrode array (3D MEA) with ten shanks, enabling functional assessment of 10 brain organoids. This approach facilitates dose‐dependent drug screening across multiple organoids. We demonstrate the effectiveness of our system through real‐time analysis of neural activity changes triggered by different doses of pottassium chloride (KCl). Furthermore, sodium channel protein type 2 subunit alpha (SCN2A)‐epileptic organoids to demonstrate utility in disease‐model‐based drug screening. Our platform enables functional screening and personalized medicine using brain organoids.

## Graphical abstract

This study presents a one‐step system that delivers different drug doses to brain organoids and monitors their neural activity using integrated 3D microelectrodes. By combining dose‐specific microfluidic delivery with high‐resolution electrophysiological recording, the platform enables simultaneous, real‐time screening of neural responses across multiple organoids—offering a powerful and scalable tool for preclinical drug testing.

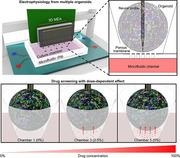

## 1 Introduction

Drug screening is a crucial process in the identification of potential therapeutic drugs prior to clinical trials. In recent years, human iPSC‐derived brain organoids have gained significant attention as a model to screen drugs for neurological diseases^[^ ^1^ ^]^ due to their structural,^[^ ^2^ ^]^ transcriptional,^[^ ^3^ ^]^ and functional similarities^[^ ^4^ ^]^ to human brain tissue. The development of disease‐model organoids, such as those for Alzheimer's^[^ ^5^ ^]^ and Parkinson's diseases,^[^ ^6^ ^]^ has further enhanced the potential of brain organoids as a tool for drug screening. However, a suitable drug screening platform that can measure functional changes by drug in brain organoids has not yet been developed.

Recently, various systems have been developed to evaluate the functionality of brain organoids by measuring neural activities.^[^ ^7^ ^]^ These systems allow for the measurement of neural activities from the surface of a brain organoid that has a 3D structure, which limits the analysis of neural activities inside the organoid.^[^ ^7b^ ^]^ Moreover, these systems lack the capability to deliver drugs and require manual administration of drug^[^ ^7b,c^ ^]^ which can only deliver a single drug to one organoid. Therefore, these systems are not suitable for high‐throughput drug screening that requires real‐time application of multiple drugs at various concentrations with measurement of neural activities.

In this study, we present an organoid‐based high throughput drug screening system for evaluating drugs from multiple brain organoids while monitoring functional change at various concentrations of multiple drugs. The system comprises a 3D microelectrode array with 10 shanks and multiple organoid culture chambers that are embedded with a microfluidic chip. The microfluidic chip includes 2 microfluidic channels and a silicon‐based porous membrane. The system allows for efficient drug screening by delivering multiple drugs at various concentrations to each of the 10 organoids in the chamber array and monitoring their changes in neural activity through the electrode array in real‐time. We demonstrate the functionality of the system by measuring the neural activities of 10 brain organoids and observing their functional changes through the delivery of KCl at various concentrations. Furthermore, we show the utility of the system through the development of an epileptic organoid that we demonstrate drug screening. This system has the potential to provide a high‐throughput and accurate evaluation of drug efficacy in various brain disease organoids.

## 2 Results

### 2.1 Design and Fabrication of the One‐Step Drug Screening System

**Figure 1:**
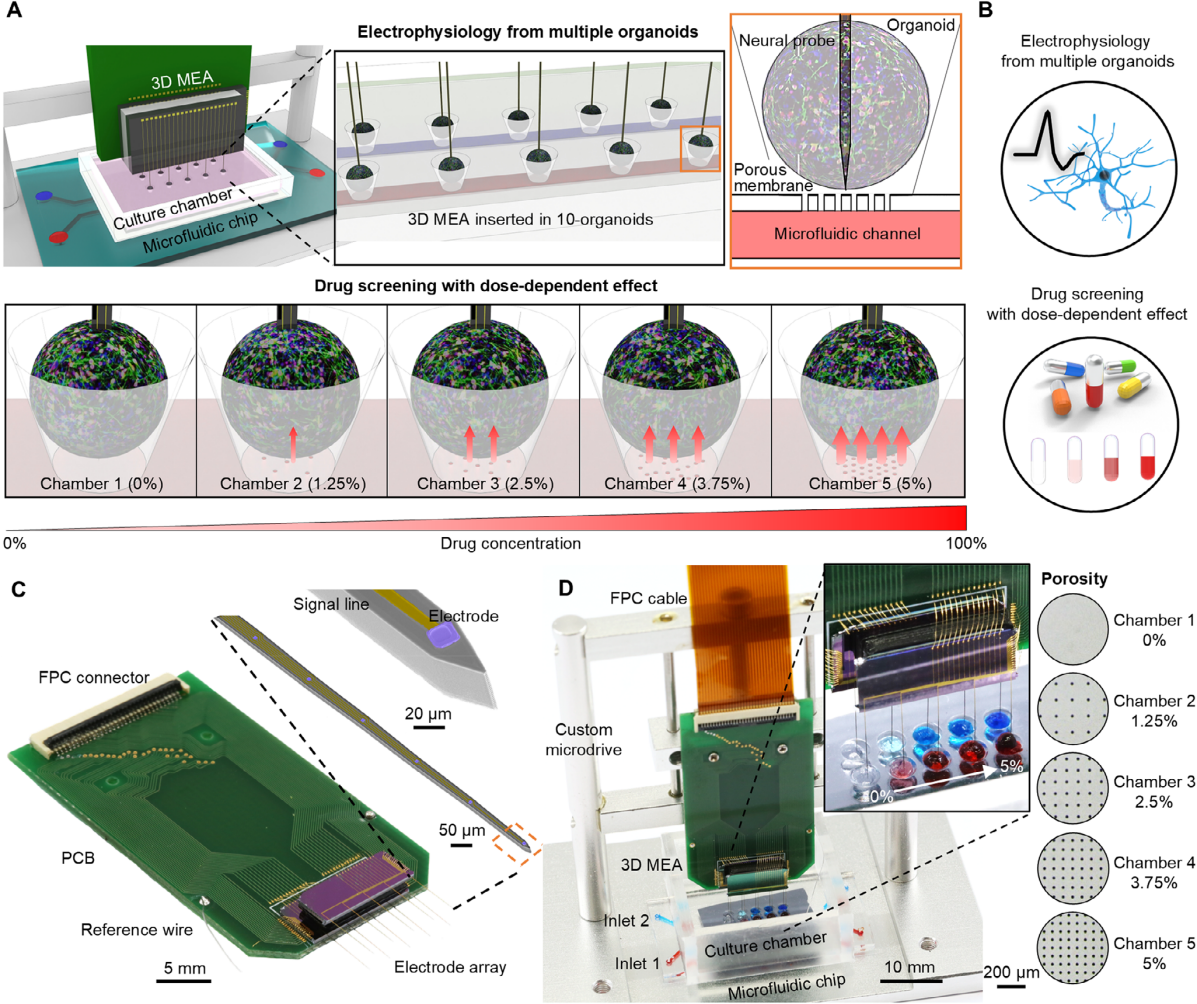
One‐step drug screening system in multiple brain organoids. A) Schematic illustrations showing structures and application to the multiple organoids. The system consists of a 3D MEA with 10 shanks and a PDMS culture chamber embedded with a microfluidic chip. 3D MEA enables simultaneous neural recording from 10 organoids and drug screening with a dose‐dependent effect. B) Schematic illustrations of the features of the system. C) Photograph showing the packaged 3D MEA. D) Photograph showing the overall system, including mechanical structure. Red and blue food dye was injected into inlets 1 and 2. The dose of food dye delivered to each chamber in the chamber was different according to the porosity of each chamber.

Our system is largely composed of a 3D MEA and a culture chamber with an embedded microfluidic chip (**Figure** **1**). The MEA, with 10 shanks, was implemented by stacking two 2D microelectrode arrays, each with 5 shanks. (Detailed fabrication and packaging processes are described in the Experimental section.) To minimize physical damage during insertion into brain organoids, each shank had a smaller dimension of 40 µm wide, 15 µm thick, and a section area of 600 µm^2^, compared to conventional neural probe^[^ ^7a^ ^]^ (145 µm wide, 40 µm thick, and a section area of 5800 µm^2^) (Figure 1C). The shank's volume only occupies 0.114% of the organoid's total volume with a 0.5 mm radius, minimizing tissue damage. The MEA also had 6 black Pt electrodes (8 µm × 8 µm) per shank, integrated with a distance of 250 µm between electrodes, allowing the measurement of neural activity from the majority of the vertical depth of an organoid (1–1.5 mm) (Figures S1, S2, Supporting Information). The shanks were 2.5 mm apart, considering the distance between each culture chamber (Figure S1, Supporting Information). Thus, we could simultaneously record the neural activities from the 10 organoids located in each culture chamber using the fabricated device.

The culture chamber consists of 2 embedded microfluidic channels and 10 small chambers integrated with a Si porous membrane on the bottom of each chamber (Figure 1D; Figure S3, Supporting Information). The chambers have an inverted trapezoidal cone shape to enable self‐alignment of the organoids at the center of the chamber. The Si porous membrane is bonded to the bottom of the chambers, with microfluidic channels integrated underneath (Figure S3, Supporting Information). Therefore, the drug injected into the microfluidic channels can be diffused into the chamber through the porous membrane. Additionally, we added a capability to control the concentration of drug delivered to each chamber by varying the membrane porosity. We designed the porosity of the five chambers on the two fluid channels differently as 0%, 1.25%, 2.5%, 3.75%, and 5% (Figure 1D). Thus, the drug was not delivered to the first chamber with the membrane porosity of 0% and a relatively large amount of drug was delivered to the last chamber with the membrane porosity of 5%. By adding this capability, our system allowed the analysis of functional changes according to the drug's concentration at once. The Polydimethylsiloxane (PDMS) culture chamber and the PDMS microfluidic chip were fabricated using a conventional soft lithography process. (Detailed fabrication process was provided in the Experimental Section.). The fabricated PDMS microfluidic channel consists of two inlet channels and two outlets, and each consists of a fluid channel with the width, length, and height of 100 µm, 35,000 µm, and 80 µm, respectively. The dimensions of the PDMS culture chamber are 25 mm × 25 mm × 10 mm, and the 10 chambers have inverted trapezoidal columnar patterns with a bottom diameter of 1 mm and a top diameter of 2 mm. Next, the 85 µm‐thick porous silicon membrane was fabricated through the etching of pores on a silicon wafer, followed by a chemical mechanical polishing (CMP) process. (Detailed fabrication methods were provided in the Methods section). The size of the pores was 10 µm, and the porosity gradually increased from 0% to 5%. At a porosity of 1.25%, 2.5%, 3.75%, and 5%, the distance between holes was 55, 39.5, 32, and 27 µm, respectively. Each component (i.e., PDMS culture chamber, Si porous membrane, PDMS microfluidic chip, and slide glass) was bonded through sequential O2 plasma bonding (100 W, 40 sec) under the microscope (Figure S3, Supporting Information). As a result, when the dye was injected through the inlet of the microfluidic channel, the different amount of drug was delivered to each culture chamber through porous silicon membrane (Figure 1D). Next, we used a custom microdrive to precisely insert the 3D MEA into 10 organoids located in each culture chamber (The detailed processes were provided in the Method section.) (Figure 1D). The microdrive allows for vertical adjustment of the 3D MEA, allowing precise insertion into the organoid.^[^ ^7a^ ^]^ Overall, our system was ready to deliver drugs while simultaneously measuring the signal changes from the 10 brain organoids.

### 2.2 Characterization of the One‐Step Drug Screening System

**Figure 2:**
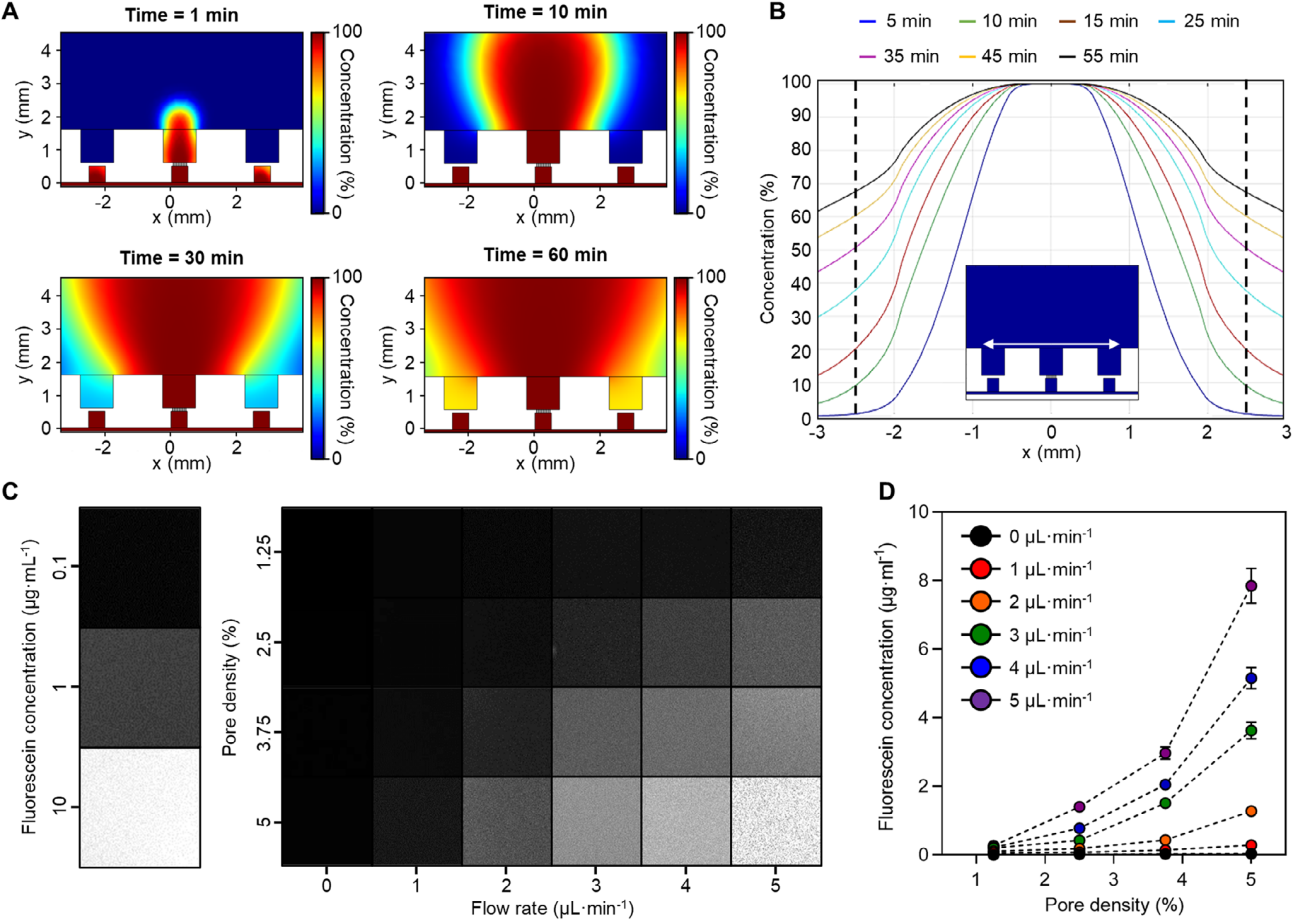
Simulation and characterization of the PDMS culture chamber embedded with a microfluidic chip. A,B) Simulation result showing drug diffusion overtime at 5 µL·min^−1^. The dotted lines in B) indicate the center position of the adjacent chamber. The white arrow in the schematic of B) indicates the *x*‐axis of the graph. C) Fluorescence images of extracted liquid samples from each chamber according to the conditions (i.e., flow rate and pore density). The left images show the fluorescence intensity according to the fluorescein concentration (i.e., 0.1, 1, and 10 µg·mL^−1^). D) Fluorescein concentration in the solution according to the flow rate and pore density. n = 3, where n is the number of samples. Data are presented as mean values +/− SD.

Before applying the system to brain organoids, we evaluated the system's performance. First, we measured the electrical impedance of the 60 black Pt microelectrodes (six electrodes on each shank). The average impedance was 45.417± 3.191 kΩ at 1 kHz, allowing the measurement of the neural activities in the brain organoids due to sufficiently low impedance.^[^ ^8^ ^]^ Next, we evaluated the performance of the capability for dose‐dependent drug delivery. First, to evaluate the interference of the delivered drug to the adjacent chamber, we estimated the convection and diffusion of the drug delivered through the porous membrane to the adjacent chambers by time through finite element model simulation (The detailed information for the simulation was provided in the Methods section). We set the cross‐sectional area for the fluidic channel for drug delivery to be the same as the actual size of the system. When the drug was delivered to the microfluidic channel at the flow rate of 5 µL·min^−1^ to a chamber with the highest porosity (i.e., 5%), we estimated the spreading of the drug according to time. We confirmed that the drug did not directly affect the adjacent chambers until ≈5 min after injection. However, 10 min after injection, the results showed that the concentration of the drug in the adjacent chamber increased by ≈10% of the concentration of the injected drug in the target chamber (**Figure** **2**A). Thus, we inferred that dose‐dependent drug delivery would be possible without affecting the adjacent chambers for up to 5 min at 5 µL·min^−1^ (Figure 2B).

Next, we evaluated the controllability of drug concentration at various flow rates. Therefore, we measured the concentration of the drug in each chamber according to flow rate (The detailed experimental protocol was provided in the Experimental Section.). To visually observe the concentration of the drug delivered to each chamber, we used fluorescein as the drug. Also, we isolated each chamber to prevent any change in the concentration of the delivered drug by continuous drug diffusion between chambers. After filling each chamber with 1 mL of DI‐water, we injected high‐concentration fluorescein (100 µg·mL^−1^) into the microfluidic channels at various flow rates for 5 min. After extracting the liquid sample from each chamber, we measured the fluorescent intensity of the extracted samples under a fluorescence microscope. Based on the fluorescent intensity according to the concentration of the fluorescein, we estimated the concentration of the drug delivered to each chamber (Figure 2C). Then, we successfully confirmed that the concentration of the drug increased as the porosity increased, and that the concentration of the drug could be controlled according to the flow rate (Figure 2D).

### 2.3 Multiple Organoid Measurements and One‐Step Dose‐Dependent Drug Delivery

First, to examine whether the geometrical properties of the inserted probes influence neural firing activity, we conducted a control experiment comparing two neural probes with different shank widths—40 µm (8‐channel) and 200 µm (32‐channel)—using cortical organoids cultured for ≈80 days (Figure S5, Supporting Information). Each organoid was placed individually in a Petri dish, and neural signals were recorded in the incubator after gentle insertion of the probe. The mean firing rate recorded with the 40 µm‐wide probe was 4.60 Hz, slightly higher than the 3.26 Hz observed with the 200 µm‐wide probe, although the difference was not statistically significant. These findings suggest that under short‐term recording conditions, the influence of shank geometry on firing activity is minimal, supporting the suitability of our MEA design for reliable neural signal acquisition.

The fabricated system was designed with two primary capabilities: 1) simultaneous recording of neural signal from multiple organoids (i.e., 10 organoids), and 2) dose‐dependent drug delivery of two kinds of drugs (Figure 1B). To demonstrate these functions, we used cortical organoids (The formation method of cortical organoids was provided in the Methods section.) (Figure S4, Supporting Information).

**Figure 3:**
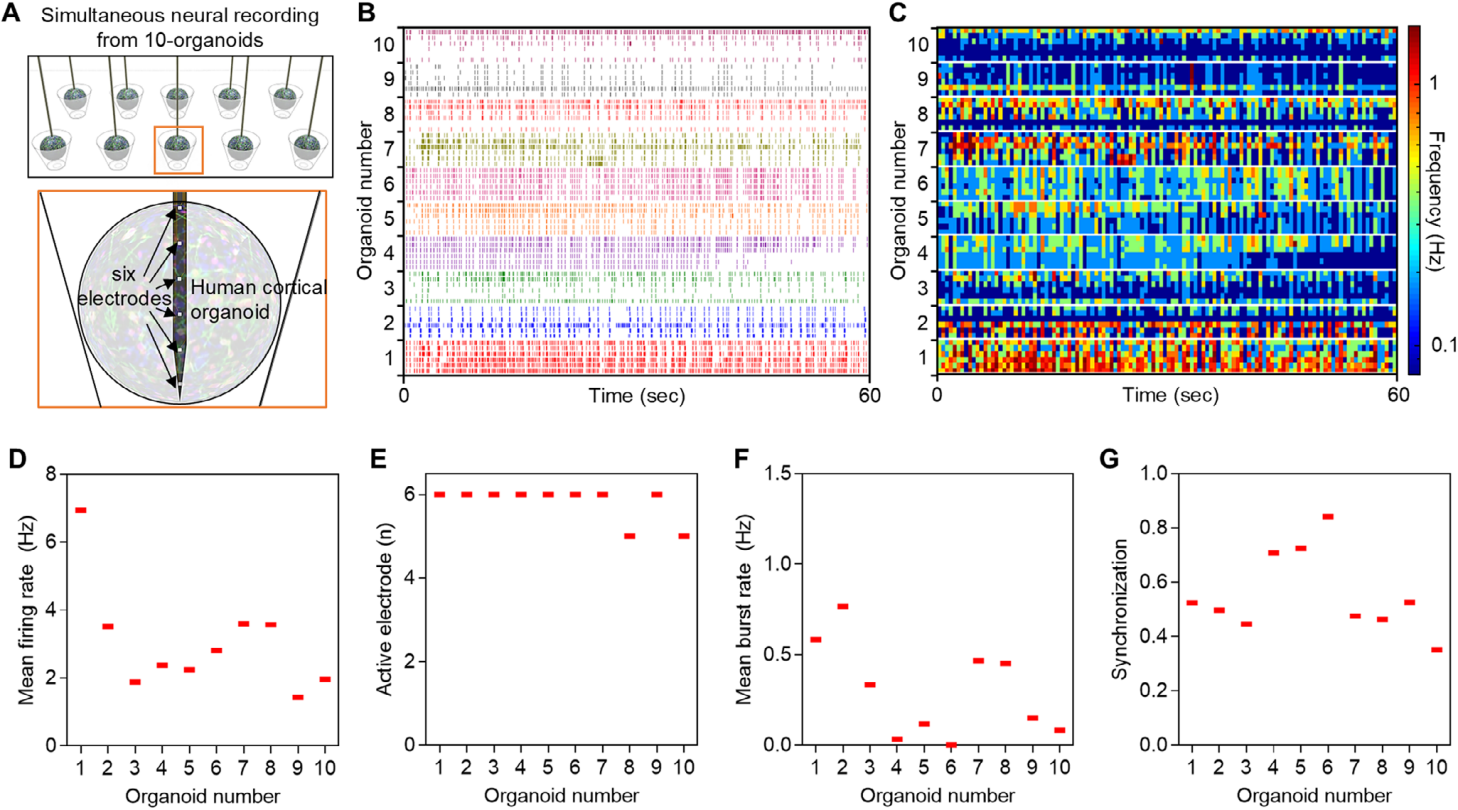
Simultaneous neural recording from 10 cortical organoids. A) Schematic illustration of the simultaneous neural recording from 10 cortical organoids. Ten shanks of the 3D MEA are positioned in each organoid. Six electrodes are positioned in the organoid. B) Raster plot showing neural spikes recorded from 10 cortical organoids. C) Color‐mapped raster plot showing neural spikes recorded from 10 cortical organoids. D–G) Graph showing functional variation between the organoids: D) mean firing rate E) active electrode number F) mean burst rate G) mean synchronized score between the electrode in each organoid.

First, to verify the capability for simultaneous signal recording, we placed the 60‐day‐old cerebral organoids in each chamber and slowly inserted the 3D MEA into each organoid (**Figure** **3**A; Figure S6, Supporting Information). Neural signals were recorded after placing the system in an incubator to maintain proper temperature and humidity. (The detailed methods for neural signal recording were provided in the Experimental Section.) We successfully measured neural signals from 10 organoids simultaneously (Figure 3B,C; Figure S6, Supporting Information). Neural signals were detected from most of the six electrodes integrated into each shank of the 3D MEA (Figure S6, Supporting Information). Notably, by simultaneously measuring multiple organoids, we could analyze functional differences in organoids of same batch or other types under the same measurement conditions (e.g., temperature, humidity, culture medium, and recording timing). Based on the measured signals from 10 organoids, we compared functional differences among the organoids (The detailed method for signal analysis was provided in the Methods section.), enabling the evaluation of variations in functional activities among organoids. The firing rate for each organoid ranged from 1 to 7 Hz (Figure 3D). Neural signals were observed in most electrodes (5 or 6 electrodes) (Figure 3E), and burst neural activity was observed in most organoids (except for number 6 of organoids). A burst rate of less than 1 Hz was noted (Figure 3F). We also analyzed synchronization between electrodes and confirmed a high synchronization score of over 40% between electrodes in all organoids (Figure 3G; Figure S7, Supporting Information). To sum up, although there are functional differences among organoids, we confirmed that all organoids were functionally mature, exhibited neural signals, and formed neural networks between matured neurons. Thus, we successfully demonstrated the system's capability to record neural signals from multiple brain organoids simultaneously and to analyze the functional differences among cortical organoids.

**Figure 4:**
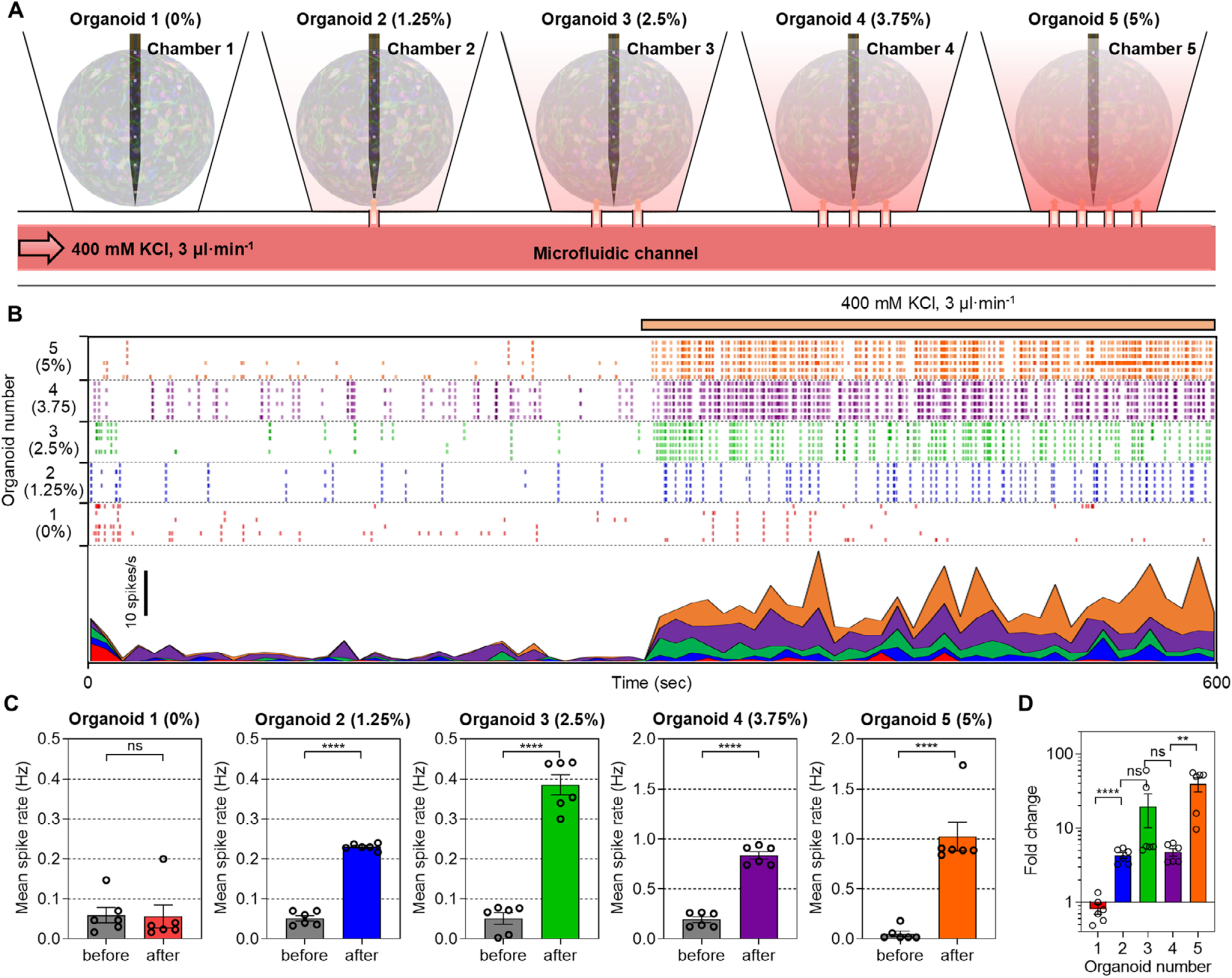
Functional changes of each cortical organoid according to dose‐dependent KCl delivery. A) Schematic illustration of the delivery of different doses of KCl to each organoid. B) Real‐time spike rate of each cortical organoid in accordance with the injection of 400 mm of KCl. The orange line above the raster plot indicates the injection duration. C) Bar plots showing the change in the mean spike rate of each organoid before and after KCl injection (n = 6 where n is the number of the electrode. Organoid 1: t(10) = 0.1012, *p* = 0.9214; Organoid 2: t(10) = 23.13, *p* < 0.0001; Organoid 3: t(10) = 11.52, *p* < 0.0001; Organoid 4: t(10) = 13.34, *p* < 0.0001; Organoid 5: t(10) = 6.712, *p* < 0.0001). D) Bar plot showing the fold change in spike rate (after KCl / before KCl) for each organoid (n = 6 where n is the number of the electrode. Organoid 1–2: t(10) = 8.602, *p* < 0.0001; Organoid 2–3: t(10) = 1.616, *p* = 0.1372; Organoid 3–4: t(10) = 1.569, *p* = 0.1476; Organoid 4–5: t(10) = 4.075, *p* = 0.0022). Data are presented as mean values +/− SD. with individual data points. All statistical analyses were performed using the unpaired two‐tailed *t*‐test. *P* < 0.05 was considered significant. ** *p* <  0.01, *** *p* < 0.001, **** *p* < 0.0001. ns: no statistical significance.

Next, to verify the system's capability for dose‐dependent drug delivery, we used KCl, which activates depolarization of the matured neurons,^[^ ^9^ ^]^ as a drug. After loading five cortical organoids in each chamber, we delivered KCl for 5 min at a rate of 3 µL·min^−1^ through the microfluidic channel (The detailed method for drug delivery was provided in the Experimental Section) (**Figure** **4**A). In the case of the organoid in the chamber with a membrane porosity of 0%, the firing rate remained constant after KCl delivery to the microchannel because the KCl was not delivered (Figure 4B,C). In contrast, the firing rate of neural signals recorded from other organoids increased after KCl delivery (Figure 4B,C). The increase in firing rate varied according to the membrane porosity of the chamber (Figure 4B,C). Specifically, the firing rate of the organoid in the chamber with a membrane porosity of 1.25% was 0.25 Hz after KCl injection, whereas the firing rate of the organoid in the chamber with a porosity of 5% was 1 Hz after KCl injection, about four times higher than the organoid in the chamber with a porosity of 1.25% (Figure 4C). In other words, the organoids located in chambers with high porosity showed higher firing rate (Figure 4B,C). Thus, we successfully delivered different doses of KCl to each chamber and confirmed that the firing rate increased according to delivered dose.

To better capture these differences, we compared neural responses using fold change (i.e., post‐KCl spike rate divided by pre‐KCl spike rate). Notably, although organoid 4 exhibited a higher absolute post‐KCl firing rate (0.836 Hz) than organoids 2 (0.23 Hz) and 3 (0.3855 Hz), it showed a smaller fold change due to its relatively higher baseline activity (0.1933 Hz), compared to the baseline rates of organoids 2 and 3 (≈0.05 Hz). This variation in baseline activity across organoids is likely due to differences in the population of active neurons captured by each probe, reflecting inherent heterogeneity among organoids. Nevertheless, we consistently observed a trend of increased firing activity with higher pore density, supporting the dose‐dependent effect of drug delivery through the porous membrane.

To confirm the reproducibility of this dose‐dependent response, we performed two additional independent experiments under the same conditions. In both experiments, we consistently observed a positive correlation between membrane porosity and the fold change in firing rate, with organoids in high‐porosity chambers exhibiting stronger responses (Figure S8, Supporting Information). However, organoids with higher baseline activity again showed relatively smaller fold increases, as observed consistently across experimental sets—for instance, organoid 3 in Experiment 2 and organoid 3 in Experiment 3 (Figure S8, Supporting Information).

To sum up, we successfully demonstrated two capabilities of the proposed system (i.e., simultaneous neural signal recording and control of drug dose delivery in each chamber delivery), enabling the evaluation of drugs at various concentrations with brain organoids.

### 2.4 Formation and Characterization of SCN2A‐Epileptic Organoids

To apply the proposed platform in screening candidate drugs for brain disease, we developed a disease organoid model from epilepsy patient‐derived iPSCs. Epilepsy is a central nervous system disorder characterized by recurrent seizures due to abnormal excessive neural activity.^[^ ^10^ ^]^ Epilepsy has been classified into several subtypes based on phenotypic patterns and underlying mechanisms.^[^ ^11^ ^]^ Although several antiseizure drugs have been approved by the Food and Drug Administration, there are no effective preclinical epilepsy models for drug testing.^[^ ^12^ ^]^ In this study, we developed an epilepsy model with brain organoids from epilepsy patient‐derived iPSCs (Figure S9, Supporting Information) to investigate the potential of our system for screening epilepsy patient‐specific antiseizure drugs.

Epileptic behaviors are known to be caused by an imbalance of excitatory and inhibitory neurons, either by activating excitatory conductance or suppressing inhibitory conductance.^[^ ^13^ ^]^ In this study, we generated cortical organoids^[^ ^14^ ^]^ using iPSCs derived from an epilepsy patient with a missense mutation in SCN2A (c.4886 G > T) (Figure S9A, Supporting Information). SCN2A encodes the voltage‐gated sodium channel Nav 1.2, which is dominantly expressed in excitatory neurons (Figure S9B, Supporting Information). ^[^ ^15^ ^]^ Mutation in SCN2A increases persistent sodium current, eventually resulting in neuronal hyperexcitability.^[^ ^16^ ^]^ We confirmed that the SCN2A‐mutated iPSCs have a normal karyotype (Figure S9C, Supporting Information) and maintain their pluripotency (Figure S9D,E, Supporting Information). We applied a cortical organoid formation method that includes excitatory neurons to create an accurate epilepsy model from iPSCs with SCN2A mutation.

**Figure 5:**
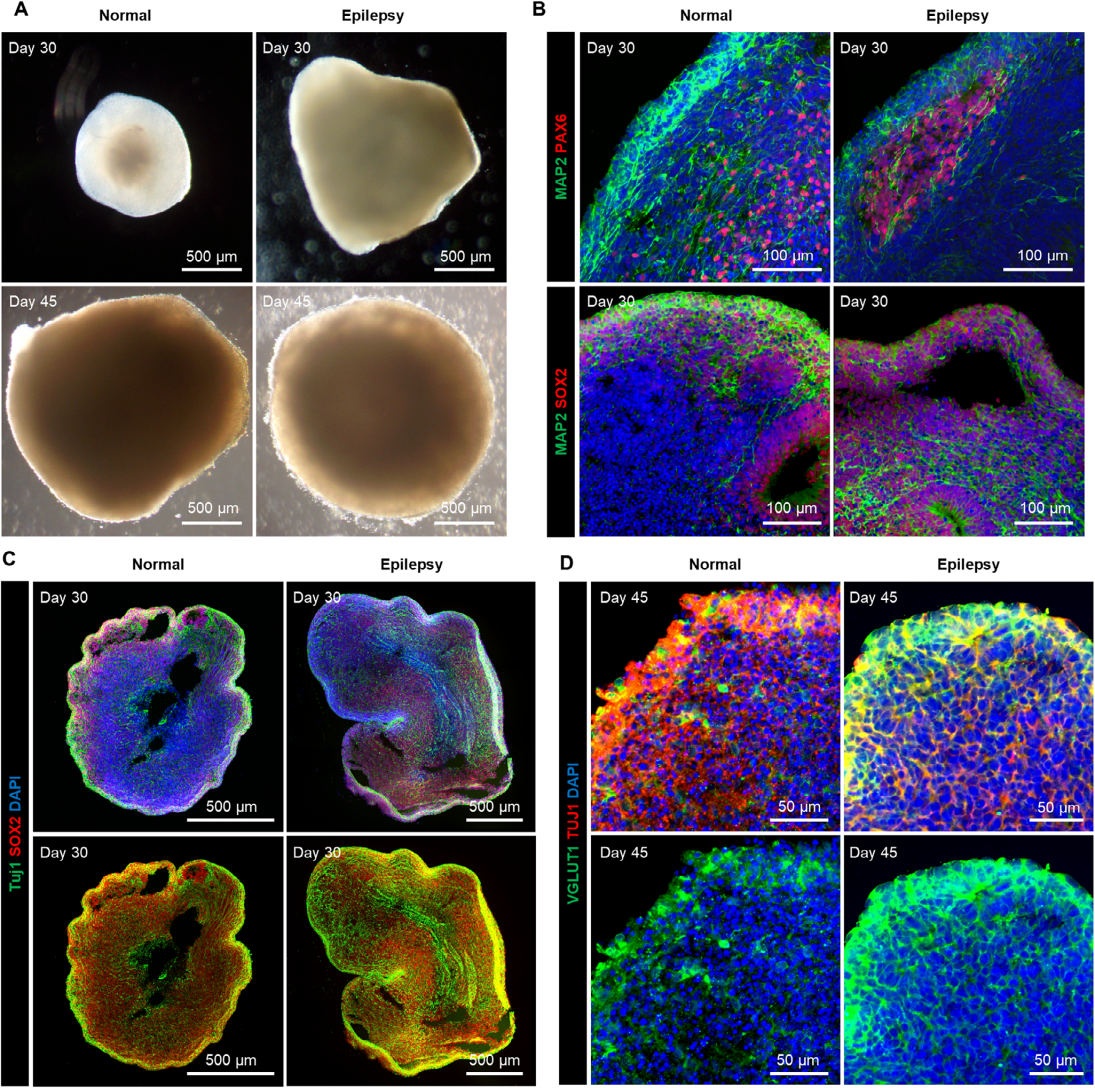
Characterization of cortical organoids derived from normal iPSCs and epilepsy patient iPSCs with SCN2A mutation. A) Bright‐field images of normal and epilepsy cortical organoids at 30 and 45 days. B) Immunohistochemical staining of neural progenitor markers (PAX6 and SOX2) and neuronal marker (MAP2) at day 30. C) The staining images of whole organoids for progenitor marker SOX2 and neuronal marker Tuj1 in normal and epilepsy organoids at day 30. D) Immunohistochemical staining for glutamatergic neuron marker VGLUT1 and Tuj1 at day 45.

To confirm the successful generation of epileptic cortical organoids from iPSCs of the seizure patient with SCN2A mutation, we compared the morphological features and neuronal marker expression between cortical organoids from normal iPSCs and SCN2A‐mutated iPSCs (**Figure** **5**A,B). Both types of organoids showed similar expression of neural progenitor markers (PAX6, SOX2) and neuronal markers (MAP2, Tuj1) at day 30 (Figure 5B,C). The marker of excitatory neurons, vesicular glutamate transporter 1 (VGLUT1), began to be expressed at day 45 in both normal and epilepsy organoids (Figure 5D), allowing seizure signals to be measured in the organoids. Therefore, electrical neural signals were measured from day 45 using our system.

**Figure 6:**
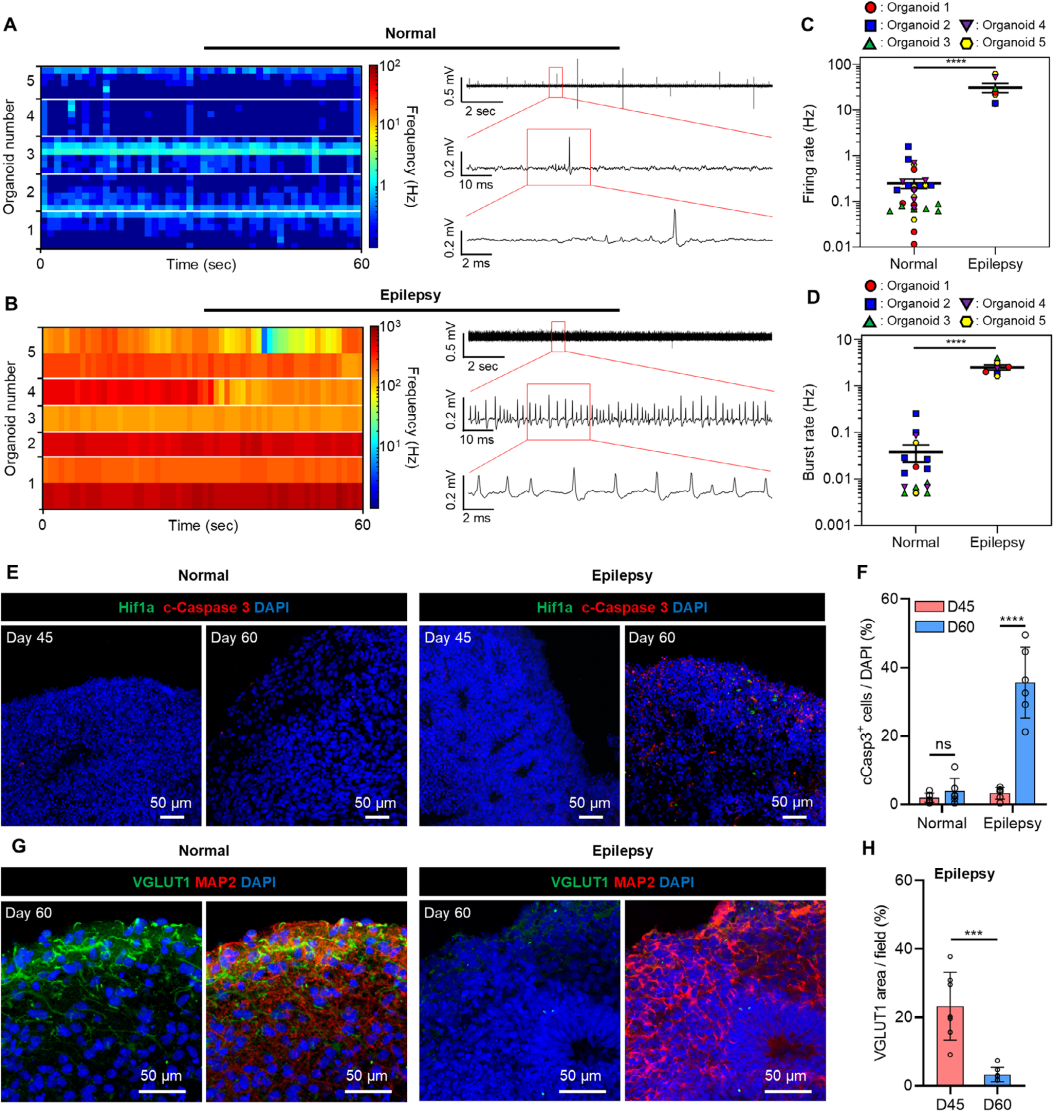
Functional properties of the SCN2A‐mutated epileptic organoid and functional comparison between normal and epileptic neural organoids. A) Color‐mapped raster plot and a transient plot showing spontaneous neural activities recorded from 5‐normal neural organoids. B) Color‐mapped raster plot and a transient plot showing spontaneous neural activities recorded from 5‐epileptic neural organoids. C,D) Dot plots showing the firing rate C) and burst rate D) measured from each electrode in normal and epileptic organoids. Only electrodes with detectable neural activity were included; electrodes that showed no measurable activity were excluded from analysis. (C: n = 30 for normal, n = 7 for epilepsy; t(35) = 9.400, *p* < 0.0001. D: n = 17 for normal, n = 7 for epilepsy; t(22) = 12.51, *p* < 0.0001). E) Immunostaining of a hypoxia marker HIF‐1α and apoptotic marker cCasp3 in normal and epilepsy organoids at day 45 and 60. F) Quantification of cCasp3^+^ cells in normal and epilepsy organoids at day 45 and 60 (n = 6 where n is the number of the organoid. Normal: t(10) = 1.490, *p* = 0.1670; Epilepsy: t(10) = 7.068, *p* = 0.000034). G) Immunohistochemical staining of VGLUT1 and MAP2 at day 45 and 60. H) Quantification of the VGLUT1‐positive area in epilepsy organoids at day 45 and 60 (n = 7 where n is the number of the organoid. t(12) = 5.212, *p* = 0.0002). Data are presented as mean values +/− SD. with the individual data point. All statistical analyses were performed using the unpaired two‐tailed *t*‐test. *p* < 0.05 was considered significant. ** *p* < 0.01, *** *p* < 0.001, **** *p* < 0.0001. ns: no statistical significance.

We analyzed the functional properties of SCN2A‐epileptic organoids using the proposed system. For comparison study, we prepared two types of organoids: 45‐day‐old SCN2A‐epileptic organoids and 45‐day‐old normal organoids. Five normal organoids were loaded in the chambers of the first row, and five epileptic organoids were loaded in the chambers of the second row. We then simultaneously recorded neural signals from the 10 organoids. A firing rate of ≈1–3 Hz was observed in normal organoids (**Figure** **6**A). In contrast, a very high firing rate of 100 Hz or more was observed in the epileptic organoids (Figure 6B). Upon zooming in on the neural signal, we observed a high firing rate similar to seizure‐like activities seen in ex vivo epileptic models^[^ ^17^ ^]^ (Figure 6B). When comparing the firing rate and burst rate, we confirmed that both were significantly higher in the epileptic organoids – more than 100 times and 50 times, respectively compared to the normal organoids (Figure 6C,D). This confirms that we successfully developed an epilepsy organoid model from epilepsy patient‐derived iPSCs.

Interestingly, in the epileptic organoids, neural signals were observed only in 1–2 electrodes located at both ends (Figure S10, Supporting Information), and synchronization between the electrodes was very low (Figure S11, Supporting Information). We inferred that necrosis in the center of epileptic organoids occurred due to increased oxygen demand caused by hyperexcitation. To prove this, we performed immunostaining for apoptosis and hypoxia markers on the epileptic organoids. Unlike normal organoids, most of the cells in the center of the epileptic organoids were dead at 45 days (Figure S12, Supporting Information). Additionally, in epilepsy organoids with the SCN2A mutation on day 60, the expression of cleaved caspase‐3 (cCasp3), a marker of cell apoptosis, was increased (Figure 6E,F), indicating that many cells underwent apoptosis. This was likely attributed to hypoxia, a characteristic of the brain tissue in seizure patients.^[^ ^18^ ^]^ Compared to normal organoids, the areas positive for a hypoxia marker hypoxia‐inducible factor‐1α (HIF‐1α) and cCasp3 were much larger in 60‐day‐old epilepsy organoids (Figure 6E,F). Interestingly, the expression of VGLUT1 decreased in SCN2A epilepsy organoids on day 60, probably due to increased apoptosis (Figure 6G,H). Overall, our data suggests that brain organoids with the SCN2A mutation provide a reliable epilepsy model undergoing neuronal development while exhibiting the pathological characteristics of seizure patients. These results show that we successfully verified the phenotype of seizures in the epilepsy organoid by measuring neural activities.

### 2.5 Drug Screening with SCN2A‐Epileptic Organoids

**Figure 7:**
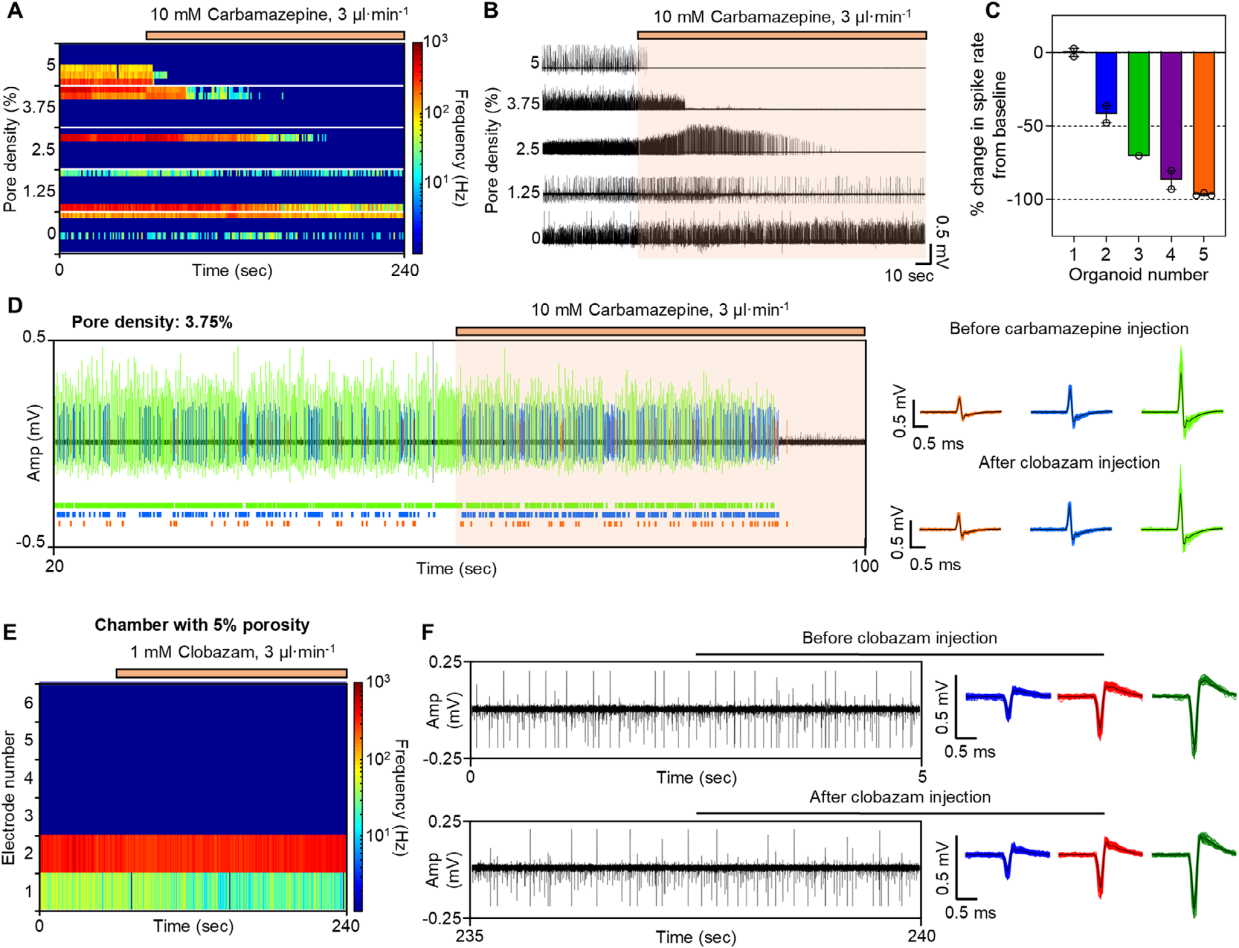
Drug screening in SCN2A‐matuated epileptic neural organoids. A,B) Color‐mapped raster plot A) and transient plot B) showing real‐time spike rate of 5 epileptic organoids in accordance with the injection of 10 mm of Carbamazepine. The orange line above the raster plot indicates the injection duration. C) Bar plot showing the change in spike rate after Carbamazepine. D) Expanded plot and spike waveforms recorded from an epileptic organoid (pore density: 3.75%) before and after carbamazepine injection. E) Color‐mapped raster plot showing real‐time spike rate of the epileptic organoid located in the hole with 5% porosity in accordance with the injection of 1 mm of Clobazam. The orange line above the raster plot indicates the injection duration. F) Transient plot and representative neural signals of the epileptic organoid before and after clobazam injection. Data are presented as mean values +/− SD. with the individual data point.

Next, we performed drug screening using the system and delivered two therapeutic agents to a 45‐day‐old SCN2A‐epileptic organoid. We chose Carbamazepine as a therapeutic agent because it is a representative treatment for SCN2A‐epileptic patients to relieve seizure‐like activity by blocking sodium channels.^[^ ^19^ ^]^ Additionally, we chose Clobazam, a highly effective treatment for SCN1A‐epileptic patients, which is expected to relieve seizure‐like activity by enhancing the post‐synaptic inhibitory effect of GABA.^[^ ^20^ ^]^ However, Clobazam is known to have no effect on SCN2A patients.^[^ ^21^ ^]^ Thus, we anticipated a decrease in seizure‐like activity with Carbamazepine but predicted minimal effect with Clobazam. When five organoids were loaded and 10 mM Carbamazepine was delivered through the microfluidic channel, we observed that the neural signal rapidly decreased, except for the organoid in the chamber with 0% porosity (**Figure** **7**A,B). Additionally, we confirmed that the drug effect was significantly enhanced as the delivered drug concentration increased according to the porosity (Figure 7C). In fact, the neural signal completely disappeared in chambers with 2.5%, 3.75%, and 5% porosity. These results indicate that the proposed system can be used to determine the optimal drug concentration. In chambers with 0%, 1.25%, 2.5%, and 3.75% porosity, we observed distinct amplitude trends following drug delivery (Figure 7): the chamber with 0% porosity showed no change, the 1.25% porosity chamber displayed a mixture of high‐ and low‐amplitude spikes, with some signals maintaining their amplitude while others gradually decreased (Figure S13, Supporting Information), the 2.5% chamber showed a transient increase in signal amplitude, and the 3.75% chamber exhibited a slight decrease (Figure 7D). These variations are likely attributable to minor mechanical shifts in the distance between neurons and the electrode during infusion, rather than pharmacological effects. In general, extracellular recordings are highly sensitive to electrode–cell proximity, with reliable signal detection typically requiring a distance of less than 100 µm.^[^ ^22^ ^]^ Even micrometer‐scale changes in this distance can lead to substantial fluctuations in signal amplitude.^[^ ^22^ ^]^ These findings also imply that drug delivery did not occur at all in the 0% porosity chamber, whereas it successfully reached the organoids in the other chambers.

As a result, we successfully suppressed the seizure‐like neural signal of SCN2A‐epileptic organoids through the application of SCN2A therapeutic agent, Carbamazepine. In contrast, when 1 mM Clobazam was delivered to the organoid in the chamber with 5% porosity, the high firing rate characteristic of seizure‐like activity persisted even after drug injection (Figure 7E,F; Figure S14, Supporting Information). This confirms that Clobazam had no therapeutic effect on SCN2A‐epileptic organoid, consistent with previously reported results.^[^ ^21^ ^]^ In summary, we successfully alleviated seizure‐like signals in SCN2A‐epileptic organoids by delivering the Carbamazepine and demonstrated the utility of the system for drug screening in neurological disease model organoids.

## 3 Discussion

The existing tools for organoids are only focused on analyzing the functionality of a single organoid. The limited capability of the tools has been a significant barrier for applications such as drug screening. To overcome this barrier, we developed a one‐step drug screening system for multiple brain organoids. Specifically, in this work, we present an unprecedented tool that integrates a 3D MEA and a drug delivery system, including capabilities for 1) simultaneous neural signal recording from multiple organoids and 2) dose‐dependent drug delivery of two drugs. The 3D MEA with 10 shanks simultaneously recorded the neural recording from 10 organoids, enabling reliable functional comparisons between different types or batches of brain organoids. Additionally, the combination of the two embedded microfluidic channels and membranes with various porosity allowed for two‐drug delivery to each organoid and observation of dose‐dependent effects.

To demonstrate the functionality of the proposed system, we successfully developed an epilepsy model organoid from epilepsy patient‐derived iPSCs. The system's application to the SCN2A‐epileptic organoids verified its high utility in reliable functional analysis and dose‐dependent drug screening. Notably, we were able to observe the real‐time functional recovery of the SCN2A‐epileptic organoids when the optimum concentration of the drug was applied. Furthermore, our platform enabled the electrophysiological demonstration of selective drug responses in SCN2A‐associated epileptic organoids—demonstrating robust suppression of neural hyperactivity by carbamazepine, whereas clobazam exhibited no comparable effect. This distinct response pattern highlights the platform's potential for personalized drug screening based on patient‐specific genetic backgrounds and functional phenotyping.

Additionally, although our inserted probe may cause minor tissue damage, it offers the distinct advantage of enabling simultaneous neural signal recording from both the interior and exterior regions of the organoid. This capability is particularly important in disease models where pathological functional changes are spatially segregated. In our SCN2A‐mutant epileptic organoids, neural signals were detected only from the outer electrodes, while the inner electrodes recorded no activity. This observation is consistent with the localized expression of caspase‐3—an apoptosis marker—predominantly in the central region of the organoids. These findings underscore the necessity of multi‐depth signal acquisition and highlight the utility of our system.

Furthermore, the proposed system supports simultaneous interfacing with up to 10 brain organoids—including both normal and disease models—enabling parallelized neural recordings. While previous platform has suggested the integration of multiple organoids,^[^ ^7b^ ^]^ none, to our knowledge, have demonstrated real‐time comparative electrophysiological measurements across multiple organoids. Our configuration significantly enhances experimental throughput and enables multiplexed comparisons between physiological and pathological states. The system's compatibility with diverse drug concentrations and its expandability further strengthens its classification as a high‐throughput platform. Therefore, our platform meets the criteria for a high‐throughput system capable of efficient and reproducible data acquisition across multiple samples under consistent experimental conditions.

Although we have realized dose‐dependent drug screening with the system in epileptic organoids, there are still some technical hurdles for utilization in other neurological disease organoids. In many neurological diseases, drug administration often requires long‐term treatment (i.e., hs to days). However, in our system, continuous drug delivery for more than 10 min causes drug interference between individual chambers, limiting the system's performance in continuously observing dose‐dependent drug effects. Consequently, the application of drugs with slow‐acting effects can be restricted. To overcome these limitations, changes in the structure of microfluidic channels are required. Specifically, applying a push‐pull fluidic structure capable of pulling the drug from the vicinity where it is injected can allow long‐term drug delivery without interference.

In addition, the observed heterogeneity in developmental state and baseline activity among brain organoids may pose limitations in interpreting pharmacological responses. Even under standardized conditions, organoid variability can arise from factors such as scaffold composition, batch differences, and initial cell density.^[^ ^23^ ^]^ For instance, Matrigel's undefined composition often leads to poor reproducibility. These factors can affect organoid maturation and baseline electrophysiological activity, introducing noise into drug‐response interpretation. To mitigate this limitation, integrating phenotypically standardized and scaffold‐controlled organoids could improve reliability. For example, recent study using silk‐based scaffolds has shown promise in enhancing reproducibility.^[^ ^24^ ^]^

Although further improvements are necessary to expand usability, our system could be a powerful tool for evaluating new therapeutic drugs using disease model organoids. Furthermore, this tool could be highly advantageous in selecting the best neurological drugs suited to a patient using patient‐specific organoids. In conclusion, we expect our system to provide new opportunities for drug screening by investigating functionality from multiple organoids and evaluating the dose‐dependent drug effects.

## 4 Experimental Section

### Fabrication and Packaging of the One‐Step Drug Screening System

The one‐step drug screening system primarily consists of two main components: (1) a 3D microelectrode array (3D MEA) with ten shanks and (2) a culture chamber integrated with a microfluidic chip.

The 3D MEA was prepared using a previously established method.^[^ ^7a^ ^]^ In brief, two planar MEAs, each containing five shanks, were individually fabricated and then aligned and assembled using a rapid adhesive process. Initially, a 400 nm‐thick silicon dioxide (SiO_2_) layer was deposited as the first passivation layer onto a 4‐inch silicon‐on‐insulator (SOI) wafer, which had a 15 µm‐thick top silicon layer. For signal line formation, thin films of titanium (20 nm) and gold (300 nm) were sequentially deposited, patterned, and etched. A second 400 nm‐thick SiO_2_ passivation layer was then deposited, and electrode areas were selectively opened by etching the oxide. Platinum microelectrodes were formed by depositing 20 nm of titanium and 150 nm of platinum onto the exposed gold regions via lift‐off. The 2D MEAs were released from the substrate using deep reactive ion etching (DRIE) on both the front and back sides of the wafer.

To construct the 3D MEA, two 2D MEAs were vertically stacked and bonded (Figure S1, Supporting Information). A plastic spacer (1.8 mm thick) was attached to the bottom MEA using a fast‐curing epoxy (401, LOCTITE) to create a total inter‐layer spacing of 2.5 mm. The second MEA layer was then carefully aligned and bonded on top under a microscope. The assembled 3D MEA was mounted onto a custom‐designed printed circuit board (PCB), and gold wires were bonded between the MEA pads and PCB using a wire bonder (Model 4526, Kulicke and Soffa Industries). The bonded wires were then protected using a biocompatible thermal epoxy (EPO‐TEK 320, Epoxy Technology, Inc.). Two flexible connectors (XF2U‐3215‐3A, OMRON) were soldered to the PCB for signal routing. A custom‐made 32‐channel flexible cable enabled electrical interfacing with additional hardware for plating or signal acquisition.

To increase the effective surface area of the electrodes, Pt‐black was electrodeposited onto the platinum microelectrodes. The plating solution consisted of 3% [w/v] hexachloroplatinic acid hydrate (HCPA), 0.025 N hydrochloric acid, and 0.025% [w/v] lead acetate in deionized (DI) water, following a standard protocol.^[^ ^25^ ^]^ Electroplating was carried out by immersing the 3D MEA, a platinum wire (counter electrode), and an Ag/AgCl wire (reference electrode) into the solution, and applying a constant potential of 0.2 V for 35 s using a potentiostat (PalmSens3, PalmSens).

The microfluidic‐integrated culture chamber consists of four stacked components: (1) a PDMS‐based culture chamber, (2) a silicon porous membrane, (3) a PDMS microfluidic chip, and (4) a bottom slide glass (Figure S3, Supporting Information). For the PDMS chamber, a metal mold was first fabricated. A 10:1 (w/w) mixture of elastomer base and curing agent (Sylgard 184 A/B, Dow Corning) was degassed, poured into the mold, cured at 80 °C for 1 h, and demolded. The silicon membrane was fabricated by DRIE patterning and etching through‐holes in a 4‐inch Si wafer to a depth of 100 µm. Afterward, the wafer was thinned by 85 µm using chemical mechanical polishing (CMP), followed by acetone cleaning.

The PDMS microfluidic chip was produced via conventional soft lithography. To fabricate the master mold, SU‐8 photoresist (80 µm thickness) was spin‐coated on a 500‐µm‐thick Si wafer, patterned, and then laser‐cut to 25 mm × 40 mm. The SU‐8 mold (20 mm × 40 mm) was placed within a metal mold cavity (20 mm × 40 mm × 5.5 mm). The PDMS mixture (10:1) was poured in, cured, and peeled off to yield a microfluidic chip (25 mm × 40 mm × 5 mm). Inlets and outlets were punched manually using a biopsy punch (Rapid‐Core – 0.05 mm, WellTech). The four layers were bonded sequentially via oxygen plasma treatment (100 W, 40 sec) using a plasma system (Covance‐MP, Femto Science).

For neural recording inside an incubator, a previously developed microdrive system and acrylic enclosure were employed.^[^ ^7a^ ^]^ The microdrive facilitated precise insertion of the 3D MEA into the organoids housed in each chamber. The acrylic enclosure minimized media evaporation and protected against contamination. To align each shank with the center of its respective chamber, the culture chamber was carefully positioned and bonded to the microdrive's base plate under a stereo microscope (SZ61, OLYMPUS).

### Simulation of the One‐Step Drug Screening System

We estimated the duration of the direct effect on the adjacent chambers by convection and diffusion using COMSOL Multiphysics 5.2 (COMSOL Inc.). For the simulation, we set the cross‐sectional area of the culture chamber embedded with the microfluidic chip to the same size as the actual size. First, the length and height of microfluidic channels were set by 8 mm and 80 µm. Also, the thickness and width of the fluid chip were set to 0.5 mm, and the height and width of the silicon pore were set to 0.1 mm and 0.01 mm. The width and height of the culture chamber were set to 25 mm and 10 mm. One end of the microfluidic channel was set as an inlet, and the other end as an outlet, and the top of the chamber was also set as the outlet. We simulated the “laminar flow” and “transport of diluted species” physics. aCSF included 145 mM NaCl in DI water delivered to the inlet at the flow rate of 5 µL∙min^−1^.

### Characterizations of the One‐Step Drug Screening System

To evaluate the electrical properties of the 3D MEA, we conducted impedance measurements on its microelectrodes. Electrochemical impedance spectroscopy (EIS) was carried out in 1 × phosphate‐buffered saline (PBS) using a saturated calomel electrode (CHI 151, CH Instruments, Inc.) as the reference. Measurements were performed with an impedance analyzer (nanoZ, Neuralynx), and impedance values of all 60 microelectrodes were recorded at a frequency of 1 kHz.

Second, the amount of drug delivered was measured to each chamber according to the flow rate from 0 to 5 µL∙min^−1^. A syringe pump (NE‐4000, NEWERA) was used to control the flow rate. Also, fluorescein (46 955, Sigma‐Aldrich) was used as a drug to estimate the amount of drug delivered. A 23‐gauge needle was connected Tygon tubing (ID: 0.5 mm, OD: 1.5 mm; S‐54‐HL) filled with the fluorescein of 100 µg∙mL^−1^ to the PMDS fluidic chip. To prevent the dilution of fluorescein delivered to each chamber by continuous diffusion, each chamber in the culture was isolated by bonding PDMS chamber array with a chamber size of 2 mm. After filling 1 mL of DI‐water in each chamber, we injected the fluorescein of 100 µg∙mL^−1^ into the inlet of the microfluidic channel at the flow rate from 0 to 5 µL∙min^−1^. After injecting it for 5 min, we extracted the DI‐water mixed with the fluorescein from each chamber. We injected each extracted sample into each microfluidic channel with 100 µm width and 80 µm height and observed the fluorescent intensity of extracted samples under a confocal fluorescent microscope (LSM 800, Carl Zeiss). Based on the fluorescent intensity according to the fluorescein's concentration, we estimated the concentration of the extracted samples.

### Generation of Human Cortical Organoids

Human cortical organoids (hCOs) were generated from human induced pluripotent stem cells (hiPSCs), which had been reprogrammed from epidermal fibroblasts. These hiPSCs were cultured on Matrigel‐coated plates (Corning, 354 277) in mTeSR1 medium (STEMCELL Technologies, 85 850). The overall differentiation process followed a previously reported protocol with minor adaptations.^[^ ^26^ ^]^

Initially, hiPSCs were dissociated into small clusters using ReLeSR (STEMCELL Technologies, 0 5872) and reseeded onto Matrigel‐coated plates in mTeSR1 medium. After two days, the maintenance medium was replaced with a neural induction medium composed of DMEM/F‐12 (Life Technologies, 11 320 033) supplemented with 1% N2 (17 502 048), 2% B27 (17 504 044), 1% non‐essential amino acids (NEAA; 11 140 050), 1% penicillin‐streptomycin (15 140 122), and 0.1% β‐mercaptoethanol (21 985 023). To promote neuroectodermal differentiation, the medium was further supplemented with SB431542 (10 µM, TOCRIS, 1614), LDN193189 (0.1 µM, Stemgent, 04–0074) and refreshed daily over a period of three days.

On the third day, the colonies were enzymatically dissociated using Accutase (STEMCELL Technologies, 0 7920) and transferred into 96‐well low‐attachment plates at a density of 5000 cells per well. The differentiation medium was supplemented with 20 ng/mL basic fibroblast growth factor (bFGF; R&D Systems, 233‐FB) and 20 ng mL^−1^ epidermal growth factor (EGF; Gibco, PHG0311). These organoids were fed daily for the next four days. Starting on day 7, bFGF and EGF were excluded from the medium to support further neural maturation.

After 8 days of culture, the developing hCOs were transferred to 60‐mm Petri dishes and maintained on an orbital shaker. From this point, organoids were cultured in a 1:1 mixture of DMEM/F‐12 and Neurobasal medium (Life Technologies, 21103‐049) containing 0.5% N2, 1% B27, 0.5% NEAA, 1% penicillin‐streptomycin, 0.1% β‐mercaptoethanol, 1% GlutaMAX (35050‐061), and 0.1 µm retinoic acid (RA). The medium was refreshed every three days to support long‐term growth and maturation.

### Generation of SCN2A‐Mutated Epileptic Neural Organoids and Normal Neural Organoids

SCN2A‐mutant and normal human iPSCs were obtained from the Yonsei University College of Medicine. The differentiation into cortical organoids was performed following the procedure outlined by Pasca et al.^[^ ^27^ ^]^

To initiate organoid formation, hiPSCs were dissociated into single cells using TrypLE Express (12 604 013, Thermo Fisher Scientific) for 7 min at 37 °C. The dissociated cells were seeded into ultra‐low‐attachment 96‐well plates (CLS7007, Corning Inc.) in Essential 8 medium (A1517001, Thermo Fisher Scientific) supplemented with 10 µM ROCK inhibitor Y‐27632 (1 293 823, Peprotech). After 24 h, the resulting embryoid bodies (EBs) were cultured in Essential 6 medium (A1516401, Thermo Fisher Scientific) with SB431542 (10 µm, Tocris Bioscience, 1614). The medium was refreshed daily during this phase.

On day 5, neural spheroids were transferred to ultra‐low‐attachment 24‐well plates and cultured in neural medium (NM), which consisted of Neurobasal‐A (10 888 022), 1% B27 without retinoic acid (12 587 010), 1% penicillin/streptomycin (15140‐122), and 1% GlutaMAX (35 050 061), all from Thermo Fisher Scientific. From day 6 to day 24, the culture medium was supplemented with 20 ng mL^−1^ of both basic fibroblast growth factor (bFGF; 4114‐TC, R&D Systems) and epidermal growth factor (EGF; AF‐100‐15, Peprotech). During this period, media changes were performed daily for the first 10 days, followed by changes every other day.

Between days 25 and 42, organoids were treated with 20 ng mL^−1^ each of brain‐derived neurotrophic factor (BDNF; 450‐02) and neurotrophin‐3 (NT3; 450‐03), both from Peprotech, to promote neuronal maturation. Starting from day 43, organoids were maintained in the same NM without additional growth factors, with media replacement every four days.

### Neural Signal Recording and Drug Delivery

Following the placement of organoids into each well of the culture chamber, the 3D MEA was gently inserted into individual organoids using a precision‐controlled microdrive. After proper insertion was confirmed, the microdrive was secured within an acrylic enclosure and transferred into a CO_2_ incubator maintained at 37 °C and 5% CO_2_ atmosphere.

Neural activity was recorded within the incubator using an RHD USB interface system (Intan Technologies), which was connected to a 64‐channel headstage linked to the packaged 3D MEA. The data acquisition was conducted using Intan's proprietary software. The recorded signals were filtered with a 300 Hz high‐pass and a 6 kHz low‐pass filter, then digitized at a sampling rate of 20 kS s^−1^ per channel.

For pharmacological experiments, a syringe pump (NE‐4000, NEWERA) was employed to deliver drugs into each chamber via the integrated microfluidic channels. After baseline (spontaneous) activity measurements, stimulation or inhibition of neural responses was performed by perfusing drug‐containing culture medium. Specifically, 400 mm potassium chloride (KCl; P3911, Sigma‐Aldrich) was used to induce excitatory activity, while 10 mM carbamazepine (94 496, Sigma‐Aldrich) and 1 mM clobazam (C8414, Sigma‐Aldrich) were administered to attenuate seizure‐like firing. The flow rate was consistently maintained at 3 µL min^−1^ for all drug deliveries. Most of the drugs were delivered to the outlet of the microfluidic channel, and a small amount of drugs was delivered into each chamber. During the drug injection, we continued measuring neural activities.

### Signal Analysis

Neural spike detection was carried out using a previously published spike‐sorting algorithm implemented in MATLAB.^[^ ^8^ ^]^ In brief, spikes were identified by applying a threshold of 75 µV, which corresponds to approximately three times the background noise level (≈25 µV). Only signals exceeding this threshold were classified as spikes and extracted for further analysis.

Detected spikes were visualized in multiple formats, including raster plots, color‐coded raster plots, and spatial electrode maps. Quantitative summaries of spike counts were presented using bar graphs. For burst detection, the ISIN‐threshold method was applied,^[^ ^28^ ^]^ using an inter‐spike interval (ISI) threshold of 0.1 s and requiring a minimum of three spikes per burst.

To assess the temporal coordination between electrodes, synchrony scores were computed using the Pyspike library, following a previously described method.^[^ ^29^ ^]^ In this analysis, spike trains recorded from different electrodes were compared: scores closer to 1 indicated high temporal alignment, while scores approaching 0 indicated low synchrony.

Functional connectivity between electrodes was visualized based on the Louvain community detection algorithm, as described in earlier study.^[^ ^7a^ ^]^ Each electrode was treated as a node, and the edge weight between nodes was determined by the calculated synchrony score. Edges with synchrony values below 0.5 were excluded to enhance clarity. Nodes sharing the same color belonged to the same functional community. Node colors ranged from blue (low synchrony, score ≈0) to red (high synchrony, score ≈1). Additionally, node size was scaled based on the number of connections, with larger nodes indicating higher connectivity.

### Characterization of SCN2A‐Mutated Patient‐Derived iPSCs

Alkaline phosphatase (AP) activity in SCN2A‐mutant iPSCs was examined using the VECTOR Red AP Substrate Kit (SK‐5100, Vector Laboratories, Burlingame, CA, USA) according to the manufacturer's guidelines. Karyotype analysis was outsourced to CancerROP Inc. (Seoul, Korea) to confirm chromosomal normality.

For immunocytochemistry, the cells were fixed with 10% formalin (HT501640, Sigma‐Aldrich) for 15 min at room temperature, followed by PBS washes. Permeabilization was performed using 0.2% [v/v] Triton X‐100 (X100, Sigma‐Aldrich) for 20 min. To reduce nonspecific binding, cells were incubated in 5% [w/v] bovine serum albumin (BSA; #216 006 980, MP Biomedicals, Santa Ana, CA, USA).

Cells were then incubated overnight at 4 °C with the following primary antibodies: anti‐SOX2 (1:1000, AB5603, Millipore), anti‐TRA‐1‐60 (1:200, MAB4360, Millipore), and anti‐OCT4 (1:100, SC‐9081, Santa Cruz Biotechnology). After washing, Alexa Fluor 488‐ or 594‐conjugated secondary antibodies (1:200; Thermo Fisher Scientific) were applied for 1 h at room temperature. DAPI (A2412, 1:1000, TCI America) was used to counterstain the nuclei.

### Immunofluorescence Staining and Imaging

To label mature neurons within human cortical organoids, the samples were fixed in 4% (w/v) paraformaldehyde (PFA) in 1 × PBS overnight at 4 °C on a shaker. After fixation, organoids were washed five times with PBS (10 min per wash) and incubated in 30% (w/v) sucrose solution at 4 °C until cryoprotected. The organoids were then embedded, frozen on dry ice, and sectioned into 40‐µm slices.

Sections were blocked for 1 h at room temperature in PBS containing 0.2% (v/v) Triton X‐100 and 3% (w/v) BSA. Primary antibodies were diluted in the same blocking solution and applied overnight at 4 °C. The antibodies included: anti‐NeuN (rabbit, 1:1000, ABN78, Millipore), anti‐MAP2 (chicken, 1:5000, AB5543, Millipore), anti‐NF‐M (mouse, 1:250, 2H3, DSHB), anti‐SATB2 (mouse, 1:200, sc‐81376, Santa Cruz), and anti‐CTIP2 (rat, 1:500, ab18465, Abcam).

After five PBS washes (10 min each), slices were incubated with secondary antibodies for 30 min at room temperature. These included Cy3‐ or Alexa Fluor‐conjugated antibodies (donkey anti‐rabbit Cy3, 711‐165‐152; donkey anti‐chicken Alexa Fluor 488, 703‐545‐155; donkey anti‐mouse Alexa Fluor 488, A21202; donkey anti‐rat Cy3, 712‐166‐150), all diluted 1:500. After final washes, sections were mounted on slides and imaged using a confocal microscope (Leica TCS SP8) with a 63 × objective.

For SCN2A‐mutant organoids, thinner cryosections (20 µm) were prepared. Permeabilization was performed with 0.2% (v/v) Triton X‐100 for 20 min, and blocking was done using a solution containing 0.02% Triton X‐100, 4% BSA (MP Biomedicals), and 2% horse serum (Thermo Fisher Scientific). Sections were incubated overnight at 4 °C with the following primary antibodies: anti‐SOX2 (Millipore, 1:1000), anti‐Tuj1 (Biolegend, 1:500), anti‐MAP2 (Cell Signaling Technology, 1:100), anti‐PAX6 (DSHB, 1:1000), anti‐VGLUT1 (Abcam, 1:200), anti‐HIF1α (Abcam, 1:500), and anti‐Caspase‐3 (Cell Signaling Technology, 1:400).

Slides were then washed three times with PBS and incubated with Alexa Fluor 488‐ or 594‐conjugated secondary antibodies (1:200, Thermo Fisher Scientific) for 1 h at room temperature. Nuclei were counterstained with DAPI (1:1000, TCI America) for 20 min. The samples were mounted using a fluorescence mounting medium (H1400, Vector Laboratories) and imaged on an LSM 880 confocal microscope (LSM 880, Carl Zeiss).

### Image‐Based Quantification

Quantification of cell death and synaptic marker expression was performed using ImageJ software (National Institutes of Health, Bethesda, MD, USA). The number of nuclei positive for cleaved caspase‐3 (cCASP3) and DAPI was manually counted from fluorescence images. For analysis of excitatory synaptic markers, the area exhibiting positive VGLUT1 staining was measured and normalized to the total image area to calculate the relative VGLUT1‐positive fraction.

### Ethical Statements

Experimental procedures involving cortical organoids from human iPSCs were approved by the Institutional Review Board (IRB) of Korea University. The use of human SCN2A‐mutated iPSCs was approved by the Institutional Review Board (IRB) of Yonsei University (Permit Number: 4‐2018‐0021).

### Statistical Analysis

All statistical analyses were performed using GraphPad Prism 8 (GraphPad Software). Data are presented as mean ± standard deviation (SD), unless otherwise noted. For comparisons between two groups, two‐tailed unpaired Student's *t*‐tests were used. The sample size (n) for each analysis is specified in the figure legends. A *p* value < 0.05 was considered statistically significant. Electrodes that did not record any neural signals were excluded from analysis. No data transformation or outlier exclusion was applied.

### Data Availability

The authors declare that all data supporting the findings of this study are available within the article and its supplementary information files or from the corresponding author upon reasonable request.

### Code Availability

The data analysis for this study was performed using Matlab 2016b and Python 3.7. The open‐source packages Py spike and python‐louvain were used to calculate synchronized scores between electrodes, which are available at https://www.github.com/mariomulansky/PySpike/ and https://www.github.com/taynaud/python‐louvain/. The Python code for the visualization of the network map used in our previous study^[^ ^7a^ ^]^ is freely available at https://www.github.com/Hyogeun‐Shin/Visualization‐of‐3D‐network‐map/tree/v1.0.0 (https://doi.org/10.5281/zenodo.4306072).

## Conflict of Interest

The authors declare no conflict of interest.

## Author Contributions

H.S. and Y.A. contributed equally to this work. H.S. and Y.A. performed most of the experiments, analyzed the data, prepared the figures, and wrote the manuscript; H.S. designed, fabricated and characterized the system; H.S. performed the electrophysiological experiments; K.‐T.K. assisted in setting up and preparing the system; Y.A. developed and analyzed the SCN2A‐epileptic organoid; J.‐H.L. and R.P. prepared the cortical organoid; J.H.K. and H.‐C.K. provided SCN2A‐mutated iPSC; W.S., S.‐W.C., and I.‐J.C. discussed the results, provided comments, and wrote part of the manuscript. All of the authors reviewed the manuscript.

## Supporting information



Supporting Information

## Data Availability

The data that support the findings of this study are available from the corresponding author upon reasonable request.
