# Supplementary material for: One‐Step Drug Screening System Utilizing Electrophysiological Activity in Multiple Brain Organoids
Source: Adv Sci (Weinh). 2025 Sep 12;12(39):e04913. doi: 10.1002/advs.202504913 (PMC12533132; doi:10.1002/advs.202504913)
Supplement: Supplementary file 1 — Supporting Information [file ADVS-12-e04913-s001.docx]

Supporting Information

One-Step Drug Screening System Utilizing Electrophysiological Activity in Multiple Brain Organoids

Hyogeun Shin^†^, Yeonjoo An^†^, Ju-Hyun Lee, Ji Hun Kim, Renuka Prasad, Keun-Tae Kim, Hoon-Chul Kang, Woong Sun, Seung-Woo Cho*, Il-Joo Cho*

^†^ These authors contributed equally to this work.

* Corresponding author.


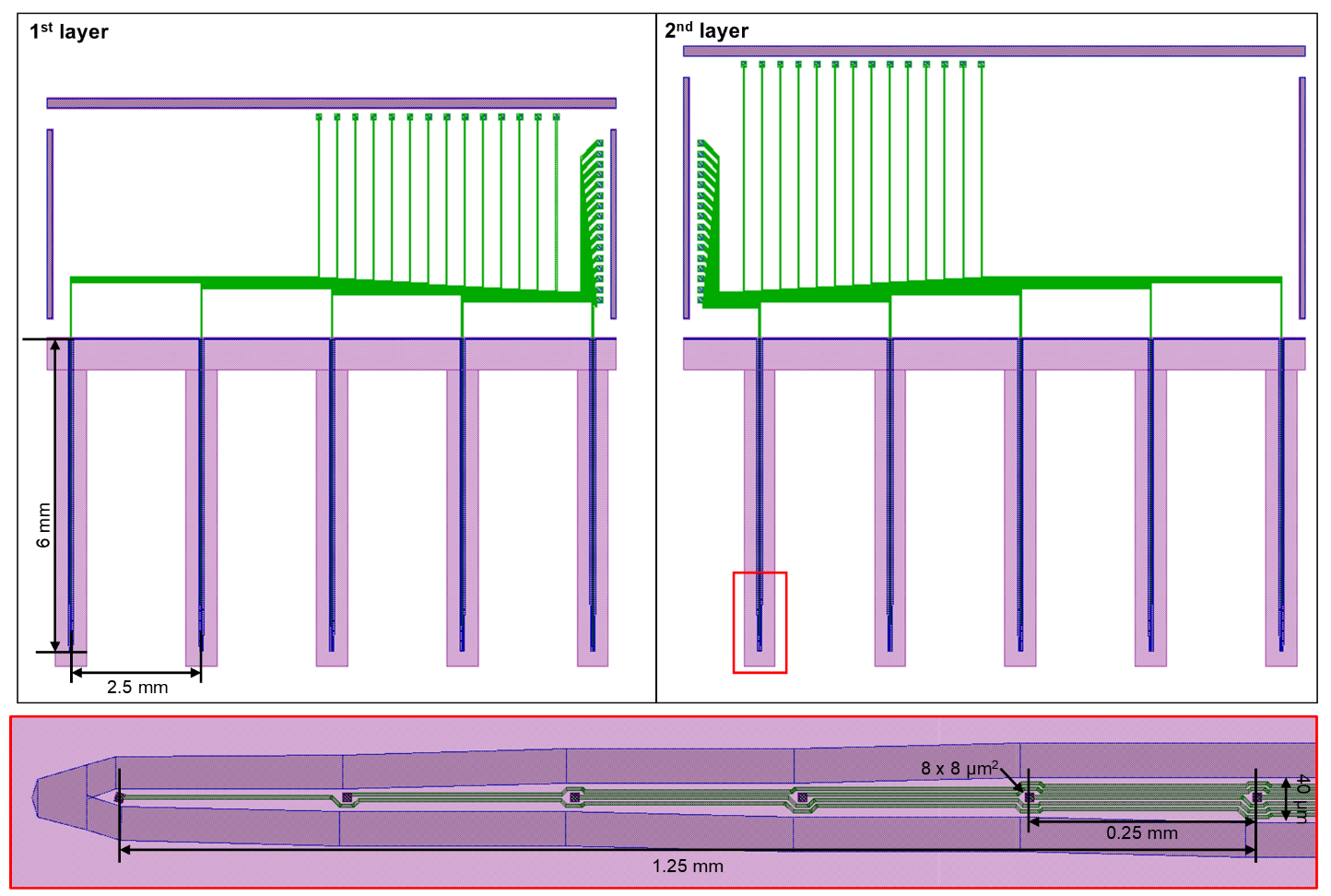


**Figure S1. Design of the 3D MEA showing the detailed dimensions.**


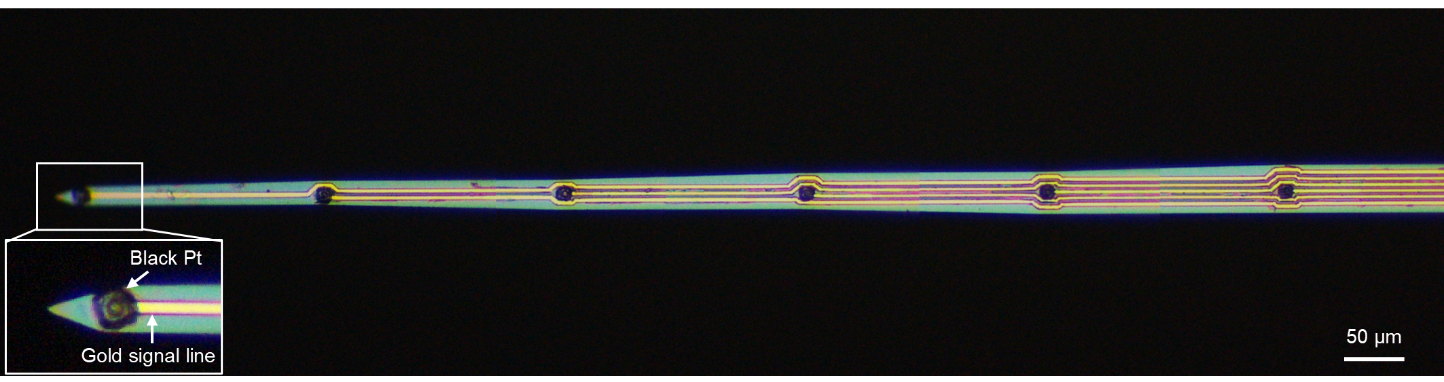


**Figure S2. Optical picture for thin one-shank of the fabricated 3D MEA.**


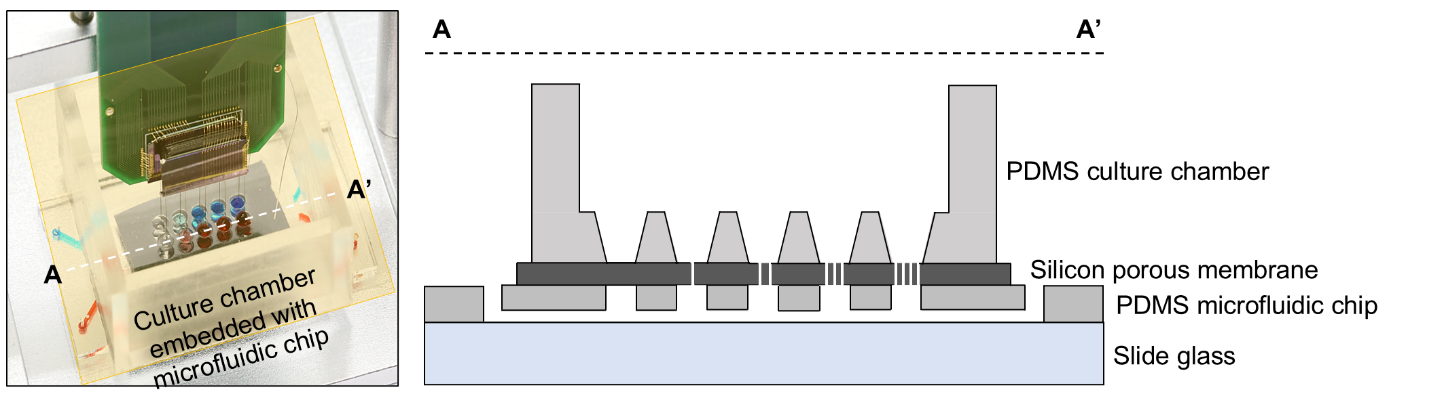


**Figure S3. Picture and cross-sectional schematic for the culture chamber embedded with a microfluidic chip.**


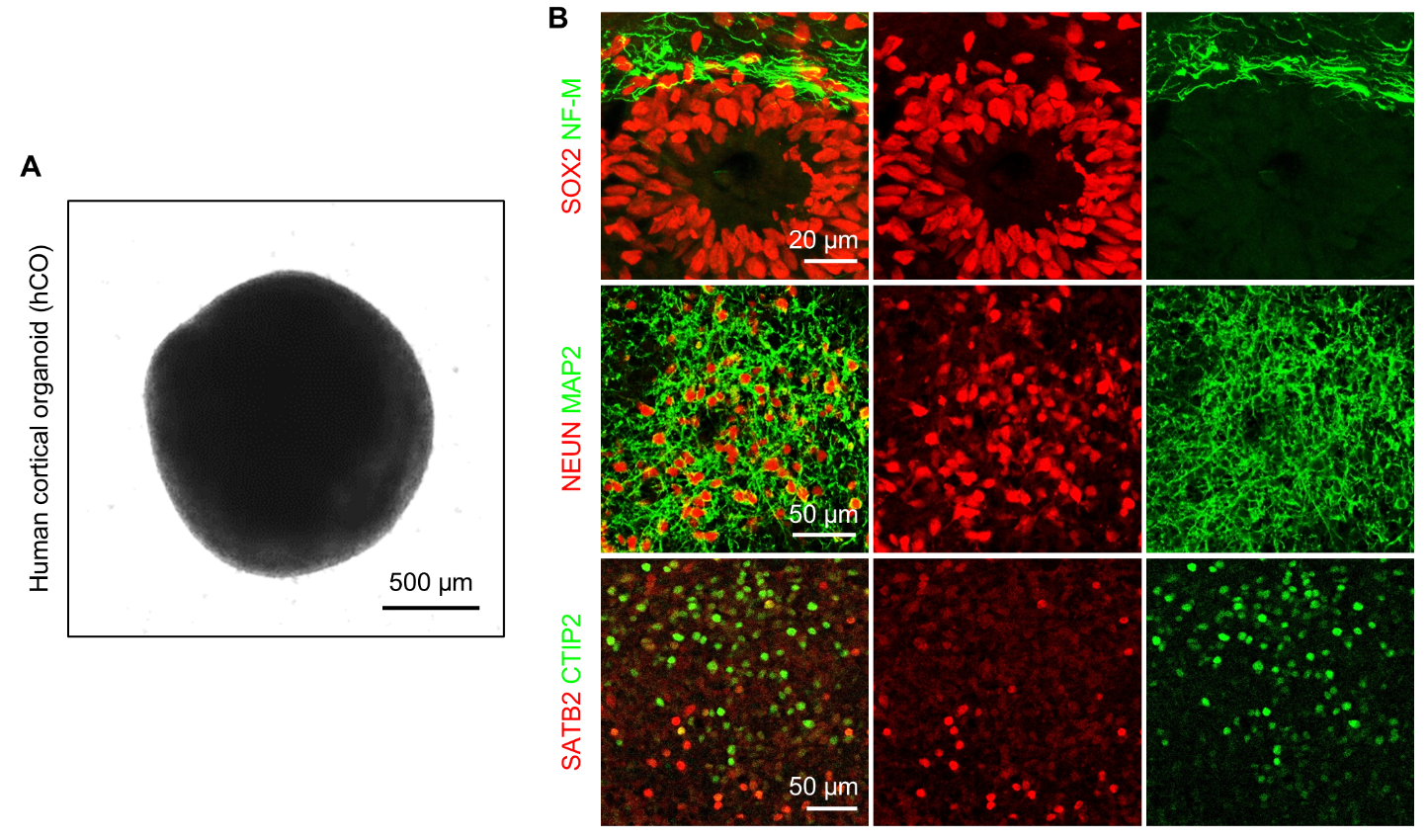


**Figure S4. Formation of human-derived cortical organoid. A,** Bright field image of 2-month-old cultured human cortical organoid (hCO) from human iPSCs. **B,** Immunostaining of 2-3 month hCOs for cortical neuron differentiation. (Top) Neural progenitor cells (SOX2, red) were radially arranged and surrounded by differentiated neurons (Neurofilament-M, green). (Middle) Differentiated neuron clusters in hCOs were visualized with pan-neuronal markers, NEUN (red) and MAP2 (green). (Bottom) Cortical layer neurons were visualized with SATB2 (superficial layer, red) and CTIP2 (deep layer, green).

**
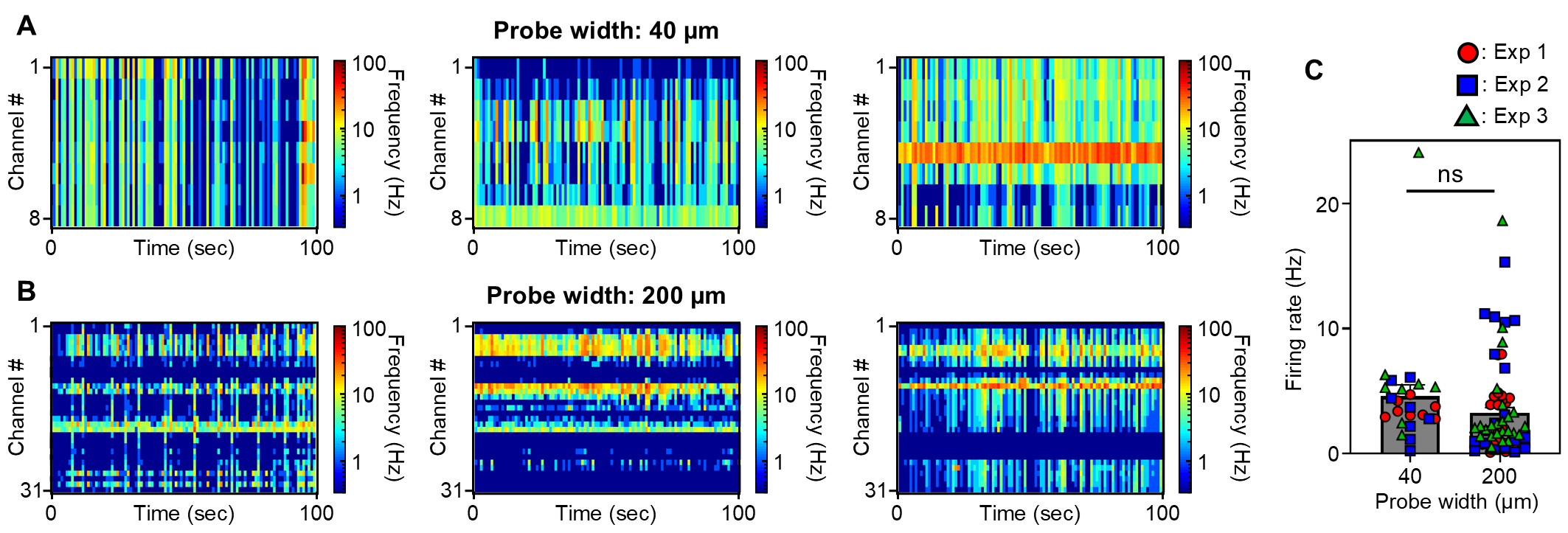
**

**Figure S5. Comparison of neural signal recordings with different probe widths in 80-day-old cortical organoids.** **A,** Representative color-mapped raster plots showing neural activity recorded using probes with a width of 50 μm (8 channels). **B,** Representative color-mapped raster plots showing neural activity recorded using probes with a width of 200 μm (32 channels). **C,** Quantitative comparison of firing rates recorded from each electrode across three independent experiments (n = 24 for 50 μm, n = 72 for 200 μm; t(94)=1.436, p=0.1542). Data are presented as mean ± s.d. with individual data points from each electrode with detectable activity. Statistical analysis was performed using the unpaired two-tailed t-test. ns: no statistical significance.


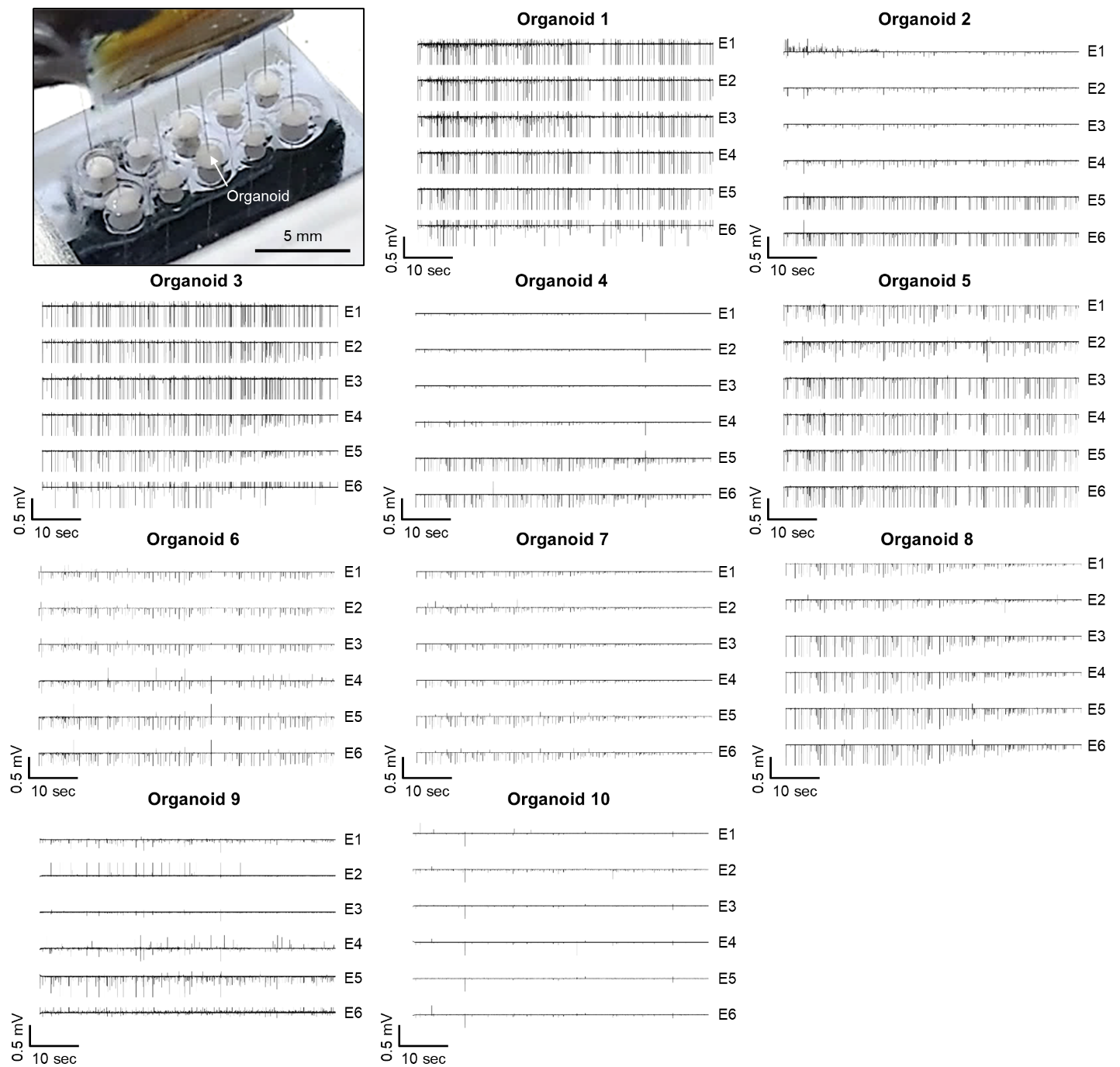


**Figure S6. Simultaneous neural recording from 10 cortical organoids.** The picture of 3D MEA inserted into 10 cortical organoids and transient plots showing recorded spontaneous activities from each organoid.


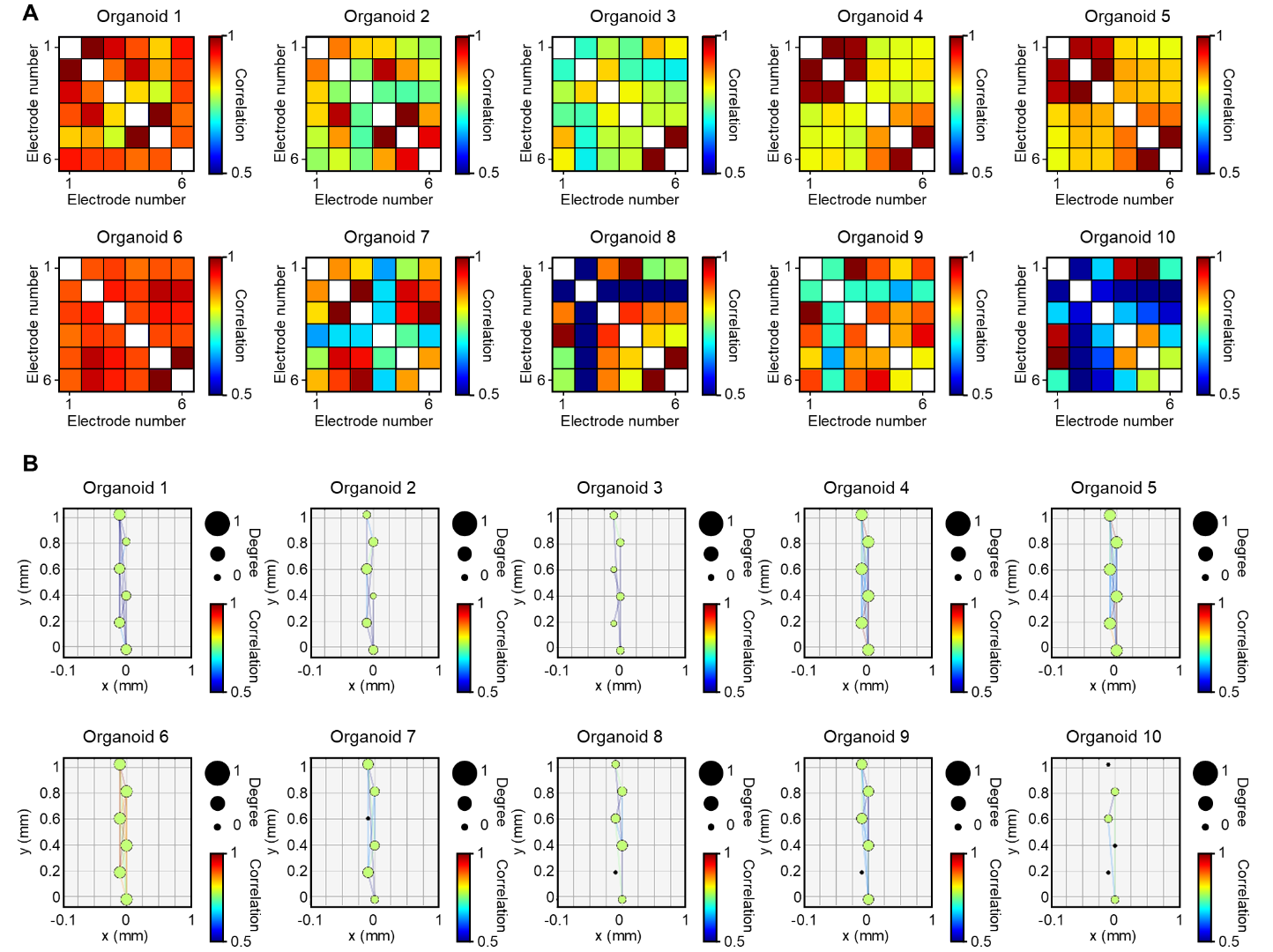


**Figure S7. Synchronization between electrodes and network maps showing connectivities among electrodes. A,** Color-mapped cross-correlation matrices showing synchronized scores between electrodes from each organoid. **B,** The color of the electrode indicates the network index connected among electrodes. The degree indicates the number of connected electrodes. The colors of the lines indicate the correlation between electrodes.

**
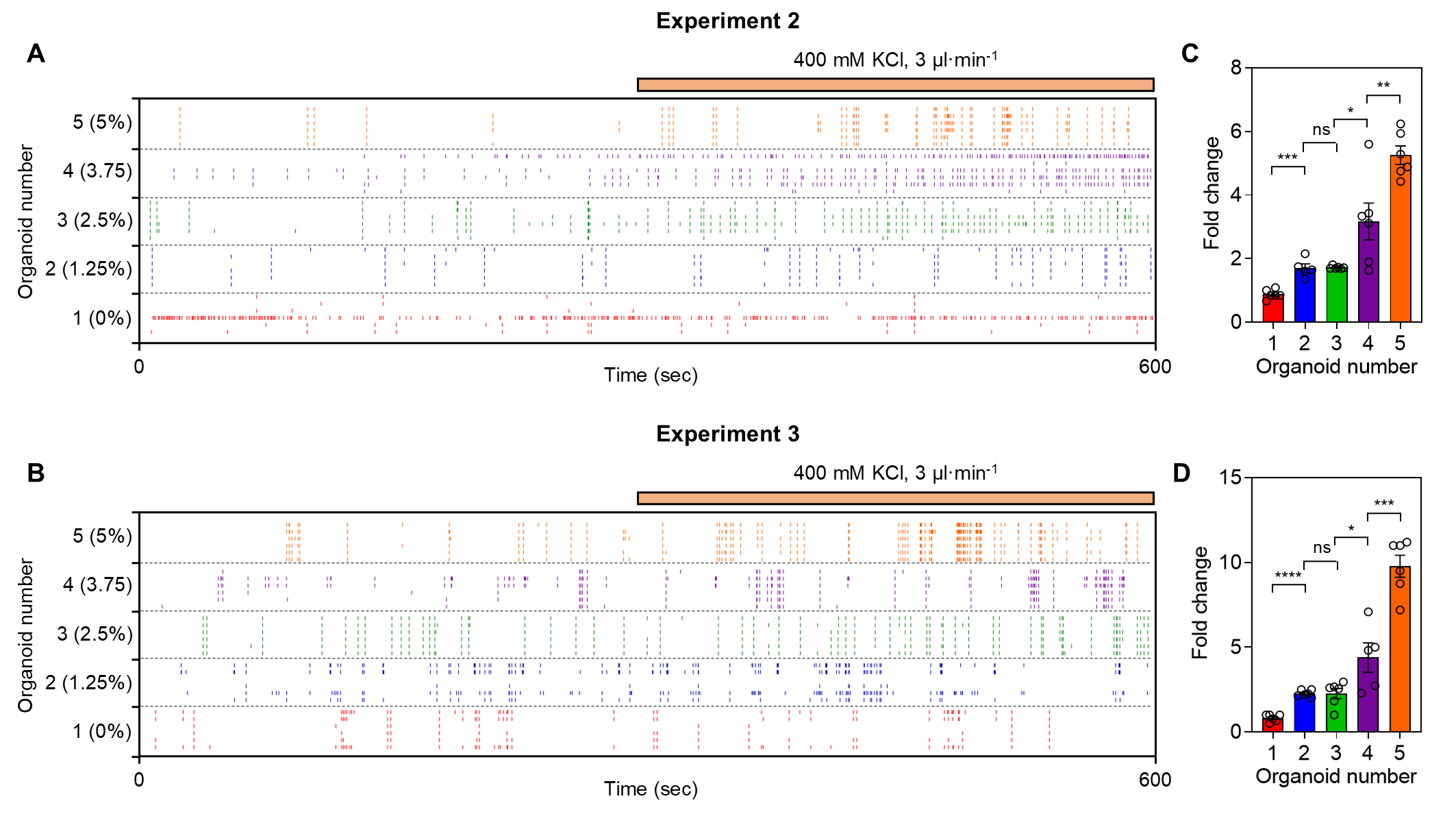
**

**Figure S8. Reproducibility of dose-dependent KCl responses across independent experiments.** **A–B,** Real-time spike rate of each cortical organoid in accordance with the injection of 400 mM of KCl in Experiment 2 (**A**) and Experiment 3 (**B**). The orange line above the raster plot indicates the injection duration. **C–D,** Bar plot showing the fold change in spike rate (after KCl / before KCl) for each organoid in Experiment 2 (**C**) and Experiment 3 (**D**), respectively (n = 6 where n is the number of recording electrodes with detectable activity, except for organoid 2 in Experiment 2 and organoid 4 in Experiment 3, which each had 5 electrodes. **C**: Organoid 1-2: t(9)=6.121, p=0.0002; Organoid 2-3: t(9)=0.1392, p=0.8923; Organoid 3-4: t(10)=2.505, p=0.0312; Organoid 4-5: t(10)=3.235, p=0.0089; **D**: Organoid 1-2: t(10)=12.11, p<0.0001; Organoid 2-3: t(10)=0.0082, p=0.9936; Organoid 3-4: t(9)=2.501, p=0.0338; Organoid 4-5: t(9)=5.070, p=0.0007). Data are presented as mean values +/− s.d. with individual data points. All statistical analyses were performed using the unpaired two-tailed t-test. p<0.05 was considered significant. ** p< 0.01, *** p<0.001, **** p<0.0001. ns: no statistical significance.


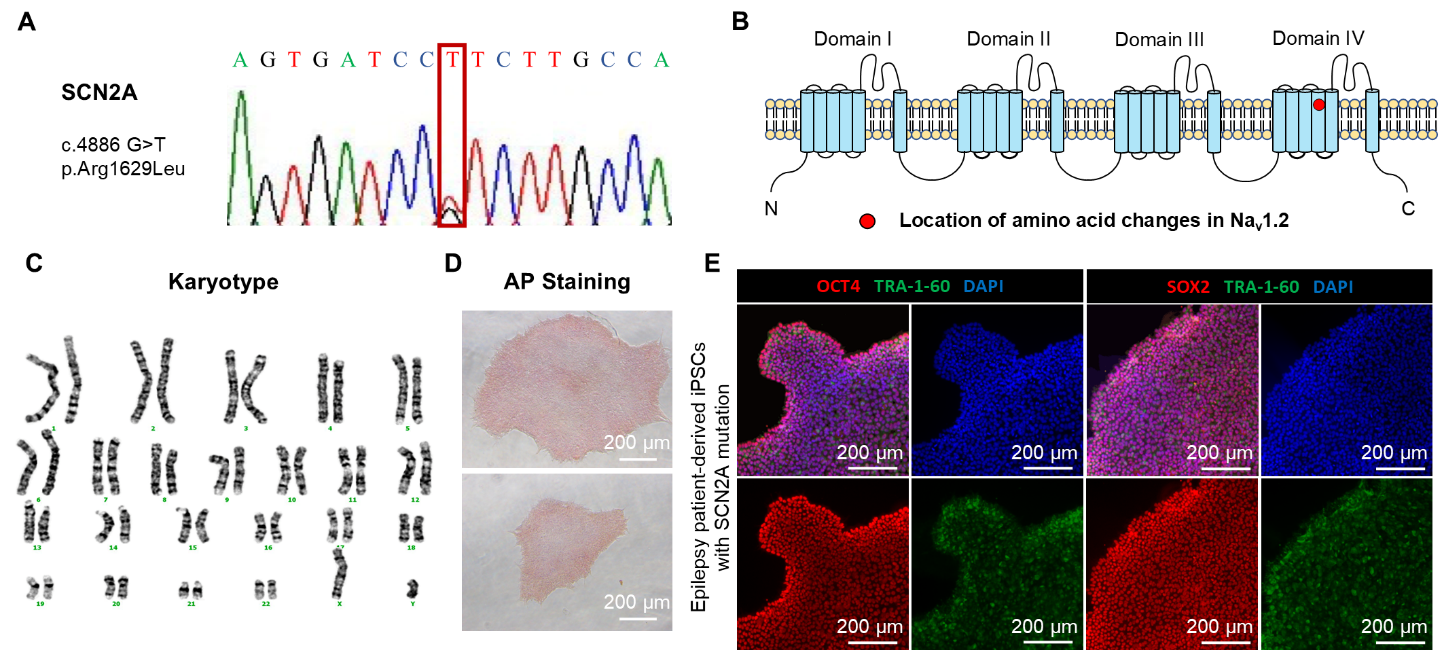


**Figure S9. Characterization of human iPSC line from epilepsy patient with SCN2A mutation. A,** Genomic sequencing to confirm the mutation in SCN2A, the presence of c.4886 G > T substitution. **B,** Schematic mutation map of the Nav1.2 channel with specific location of amino acid change. **C,** Karyotyping of epilepsy iPSC line to confirm a normal human male karyotype (46, XY). **D,** Alkaline phosphatase (AP) staining images of epilepsy patient iPSC line. **E,** Immunofluorescent staining for several pluripotency markers (TRA-1-60, OCT4, and SOX2).


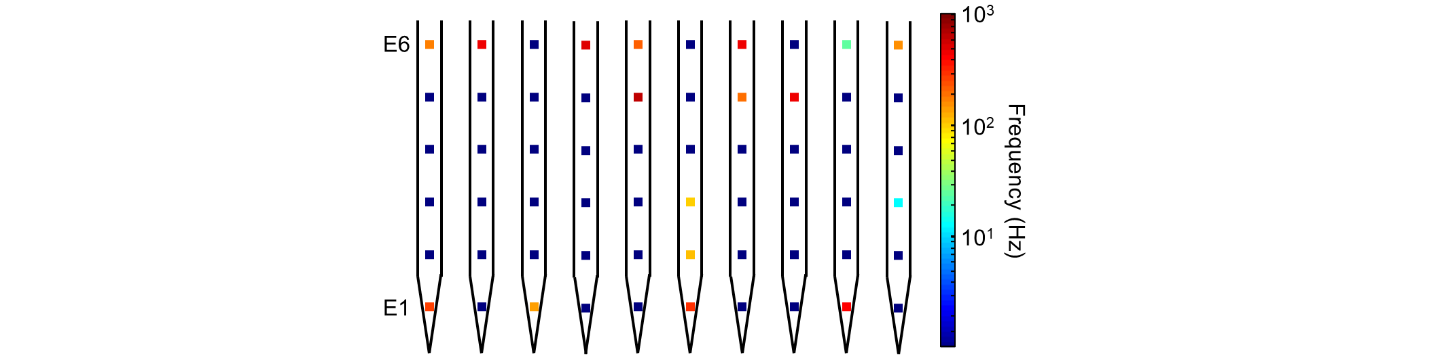


**Figure S10. Visualization of the spike rates recorded from each electrode in 10 epileptic neural organoids.** Seizure-like activities were measured at the edges of the organoids (e.g., E1 and E6).


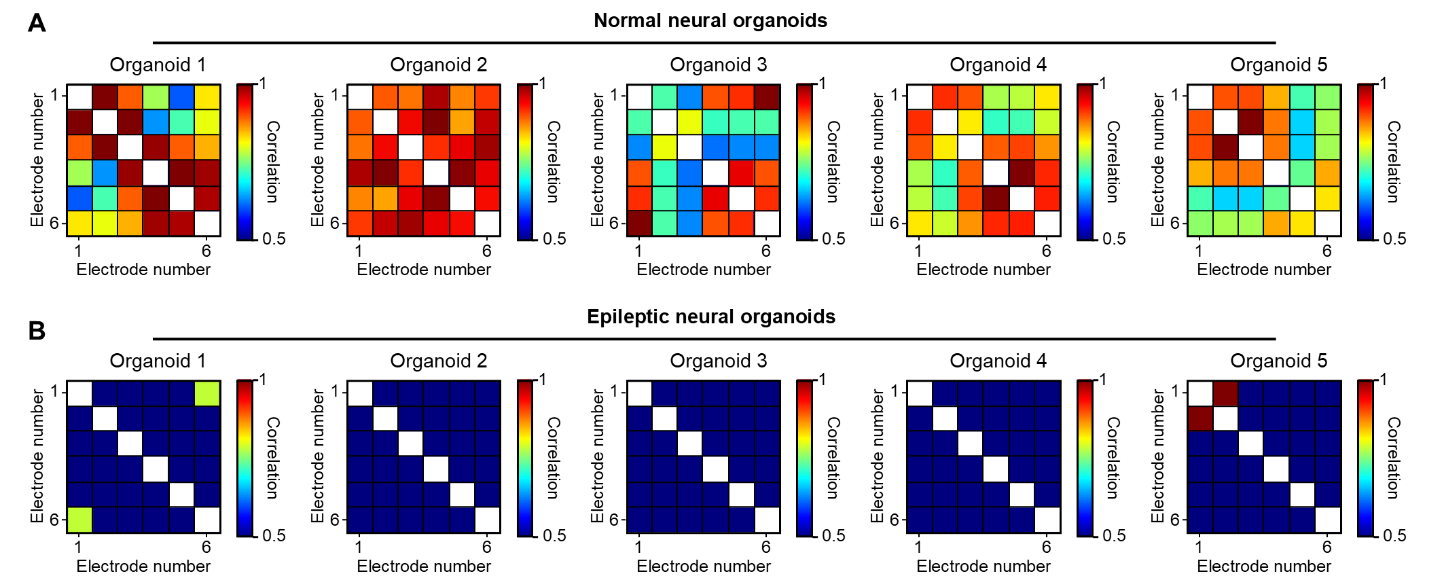


**Figure S11. Color-mapped cross-correlation matrices showing synchronized scores between electrodes from normal and SCN2A-epileptic organoids.**


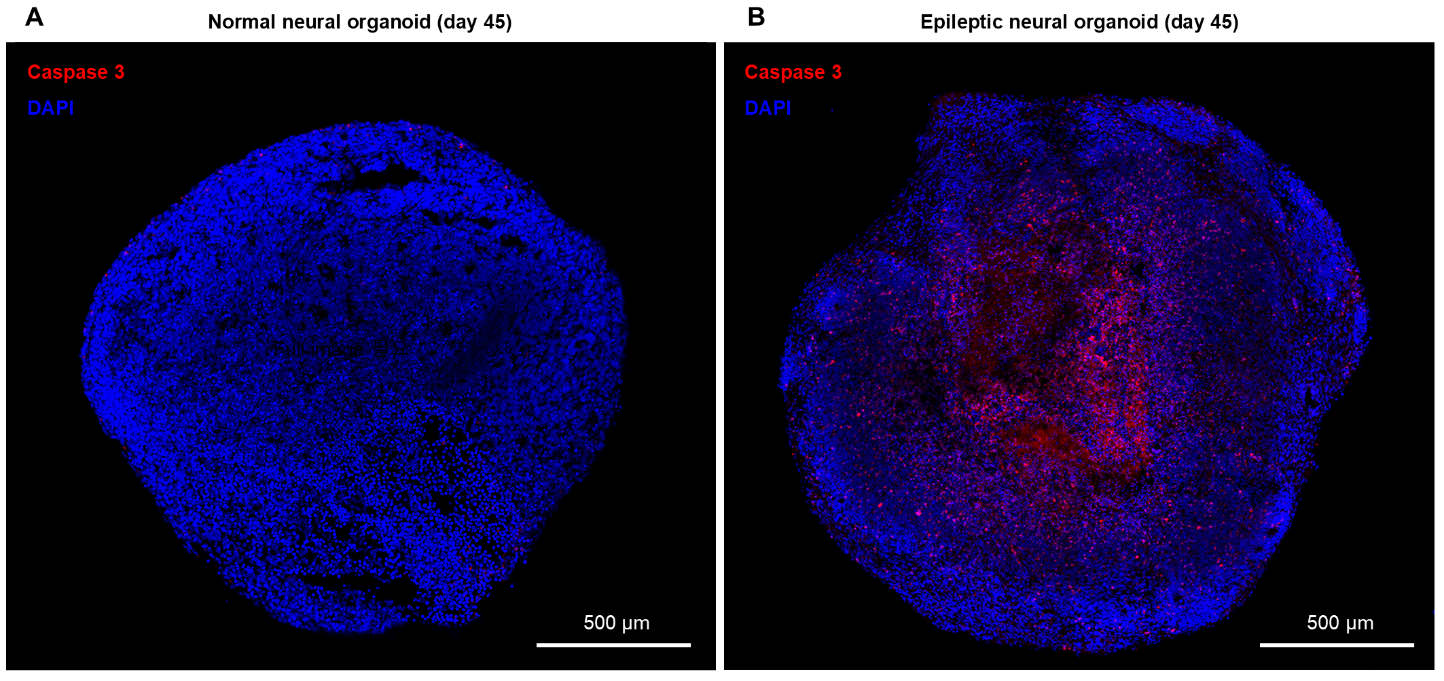


**Figure S12. Comparison of cCasp3+ cells population in (A) WT and (B) epilepsy organoids at day 45 based on the immunostaining images of whole organoids.**


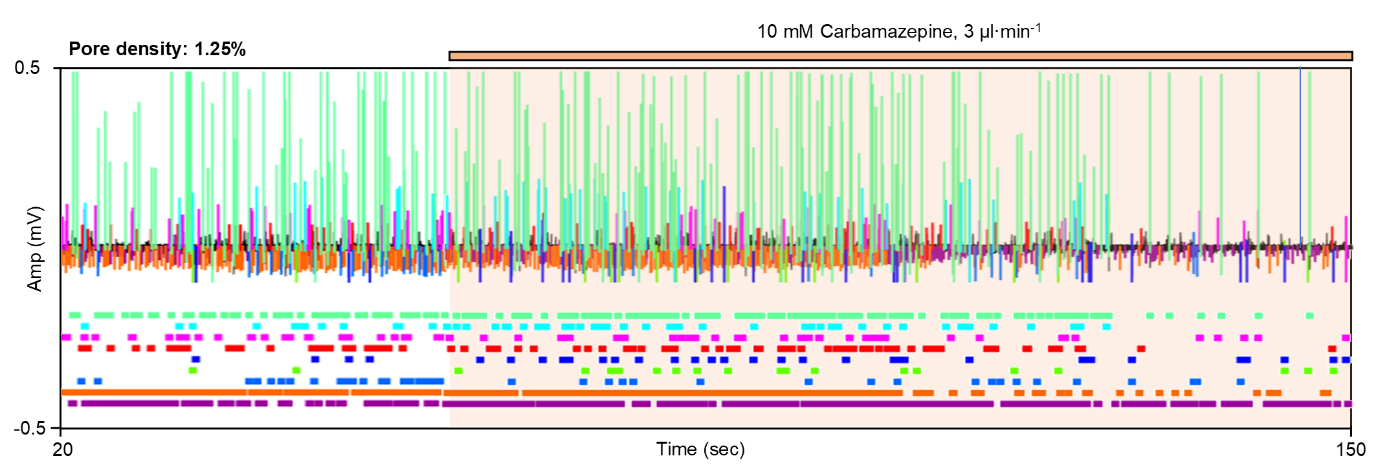


**Figure S13. Expanded spike trace from a chamber with 1.25% pore density during carbamazepine infusion.** The green trace maintains large-amplitude spikes throughout the recording, while the orange trace shows a gradual reduction in amplitude following drug delivery.


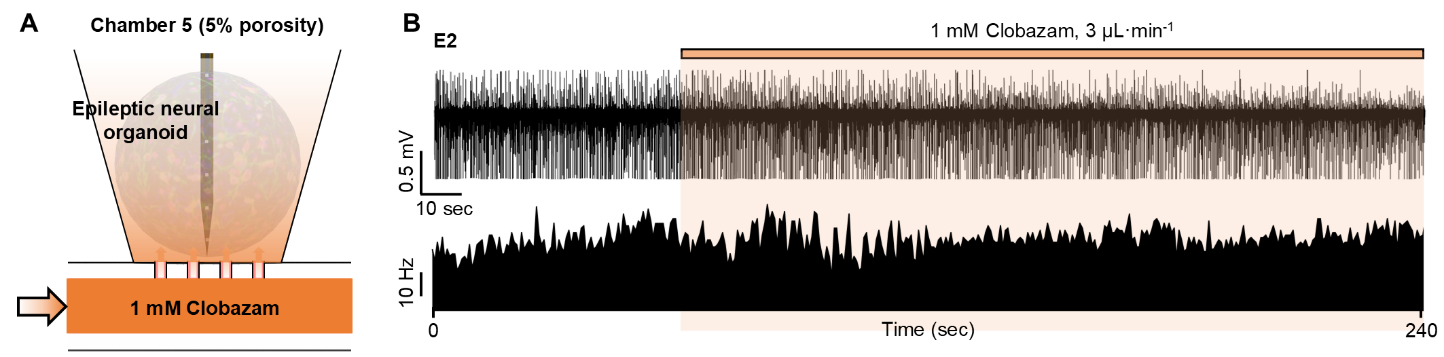


**Figure S14. Changes in seizure-like activities by 1 mM Clobazam delivery into the microfluidc channel. A,** Schematic illustration of the delivery of 1 mM Clobazam to epileptic organoid in the chamber with 5% porosity. **B,** Transient plot and real-time spike rate before and after 1 mM Clobazam delivery.
